# Answering medical questions in Chinese using automatically mined knowledge and deep neural networks: an end-to-end solution

**DOI:** 10.1186/s12859-022-04658-2

**Published:** 2022-04-15

**Authors:** Li Zhang, Xiaoran Yang, Shijian Li, Tianyi Liao, Gang Pan

**Affiliations:** 1 Advanced Technology Research Institute, Zhejiang University, Hangzhou, China; 2 Department of Computer Science, Zhejiang University, Hangzhou, China

**Keywords:** knowledge graph, Question-answering system, Text mining, Bootstrapping, Deep learning, Memory neural network, Natural language processing

## Abstract

**Background:**

Medical information has rapidly increased on the internet and has become one of the main targets of search engine use. However, medical information on the internet is subject to the problems of quality and accessibility, so ordinary users are unable to obtain answers to their medical questions conveniently. As a solution, researchers build medical question answering (QA) systems. However, research on medical QA in the Chinese language lags behind work on English-based systems. This lag is mainly due to the difficulty of constructing a high-quality knowledge base and the underutilization of medical corpora in the Chinese language.

**Results:**

This study developed an end-to-end solution to implement a medical QA system for the Chinese language with low cost and time. First, we created a high-quality medical knowledge graph from hospital data (electronic health/medical records) in a nearly automatic manner that trained a supervised model based on data labeled using bootstrapping techniques. Then, we designed a QA system based on a memory-based neural network and attention mechanism. Finally, we trained the system to generate answers from the knowledge base and a QA corpus on the internet.

**Conclusions:**

Bootstrapping and deep neural network techniques can construct a knowledge graph from electronic health/medical records with satisfactory precision and coverage. Our proposed context bridge mechanisms perform training with a variety of language features. Our QA system can achieve state-of-the-art quality in answering medical questions with constrained topics. As we evaluated, complex Chinese language processing techniques, such as segmentation and parsing, were not necessary for practice and complex architectures were not necessary to build the QA system. Lastly, we created an application using our method for internet QA usage.

**Supplementary Information:**

The online version contains supplementary material available at 10.1186/s12859-022-04658-2.

## Background

Medical information is essential and has thus rapidly increased on the internet, constituting a significant proportion of network searches. As reported, one-half of American adult netizens search for medical information [1]. More than one-half of queries in search engines concern medical care in China [2]. However, the quality and accessibility of medical information on the internet are insufficient [3, 4]. In particular, the reliability and accuracy of the results are not high, and a large amount of information is misleading or pertains to advertising [5]. In addition, some results are difficult for ordinary users to understand without sufficient expertise [6, 7].

Medical question answering (QA) systems have become a popular research topic in medical information services due to their ability to find answers in data for users’ questions in their native language. Although many medical QA systems succeed in efficiently providing information to users [7], there has been little research on systems for the Chinese language. As discussed below, building a medical QA system for the Chinese language faces several challenges.

### Difficulty of building knowledge bases for Chinese medical QA

Current QA systems combine a knowledge base (KB) with statistical methods, significantly improving QA results by searching entities and relations based on a user’s query, such as IBM Watson, Google Now, and Apple Siri [8–10]. However, research on Chinese-language-based medical KB research is limited [11–13]. The construction of a medical KB in Chinese requires considerable time and labor, as it must cover tens of thousands of concepts and relations while maintaining high accuracy and usability [11].

### Underutilization of medical corpora in Chinese

To build a KB and train a QA system,we need to exploit medical texts in Chinese. More specifically, it is necessary to extract semantic information from a text and recognize semantic entities and relations, which requires an annotated corpus for training. Chinese words exhibit extensive semantic ambiguity [14, 15], and the problem worsens because the Chinese language does not have spaces [1]. Moreover, Chinese electronic health/medical record (EHR) corpora are not written with accurate grammar and are often oversimplified, omitting many language structures. Consequently, few effectively trained parsers for Chinese medical corpora and few Chinese-language-based medical KBs.

### Complexity of adopting KB in QA systems

Existing KB-QA systems often rely on a complex inference mechanism to use knowledge in QA. Semantic parsing techniques [16–18] often involve complex processes of question processing, document semantic matching, and answer selection. Text embedding techniques that transfer text into vectors make it possible to build end-to-end QA systems without parsing models [19–21]. However, there has not been sufficient research to indicate which techniques and architectures are necessary to build a KB-QA system that learns to answer medical questions from medical corpora written in irregular and oversimplified Chinese.

We believe that the key to solving the challenges in medical QA in the Chinese language is to build a high-quality medical knowledge graph, which can help semantic processing and improve QA matching results. In this paper, we propose an end-to-end method that automatically constructs a medical knowledge graph and exploits the graph in a deep-learning-based QA system. We designed this method to save both patients’ and providers’ time and cost in information searching and servicing, respectively. The main contributions of this study are as follows:

1. We constructed an automatic semantic annotation method based on bootstrapping technology that tags medical entities and relations in Chinese medical electronic records.
2. We automatically constructed and trained a model to build a knowledge graph via semantic extraction. The model extracts medical entities and relations from raw text and merges them into a knowledge graph with entity alignment and consistency analysis.
3. We constructed a QA system based on deep neural networks (DNNs; attention- and memory-based) that can answer native-language healthcare questions for a single disease, treatment, symptom, and test. In addition, we wrapped this system into an application for end-users.

### Related works

#### Research on medical QA systems

Medical QA is receiving increasing attention from researchers and enterprises. In 2017, the Text REtrieval Conference (TREC) started a competition for medical QA [22]. Current medical QA systems mainly use two techniques: statistical and KB methods.

Statistical methods train a model from a QA corpus, match new questions to historical questions, and retrieve the corresponding answers from the stored documents. Typical statistical medical QA systems are AskHermes and HONQA [23]. These systems are easy to train; however, the results are old documents, and their accuracy (precision and recall) and answer format are not optimized.

In contrast, KB-QA systems can analyze semantics in documents and user queries, provide precise results, infer user intentions, and synthesize user-friendly answers. Typical medical KB-QA systems are MedQA [7] (an early method for facts) and MEANS [12] (a new method for multiple questions). These KB-QA systems are mainly designed for the English language and based on mature medical knowledge bases/tools, such as MetaMap [24].

Research on medical QA systems in Chinese is relatively limited [25], and studies on knowledge-based QA for Chinese medical questions have not reported promising results. Liu et al. [11] conducted a study where they first annotated structured web pages and used text mining algorithms to form an essential medical KB in Chinese for answer searching. However, they did not carefully assess the performance of their system. Their study did not present an acceptable accuracy; thus, the KB mined from the internet may suffer from poor quality. Liu et al. conducted a more solid study [13]. In their study, they manually built a medical KB; however, they only used the KB for oversimplified QA tasks and did not demonstrate the advantages of the KB. Recently, He et al. [1] proposed an end-to-end solution for medical QA in Chinese. They created a sufficiently large QA training corpus, adopted a deep learning matching model, and achieved higher than 60%. This study is different in terms of the technical route; namely, we attempted to combine a KB and deep learning model to generate answers in an end-to-end manner. The KB is transferred into embedding vectors in our system and stored with recent attention-based and memory-based DNNs with an appropriate design for the Chinese-language medical QA task. From our experiments, the precision and recall demonstrate the usability of our proposed system for ordinary internet users for simple-fact medical QA.

#### Research on mining a KB

Several authors have reported that the construction of medical QA systems is subject to difficulties in constructing a medical knowledge graph, as structured data are relatively rare. They also proposed methods to obtain medical knowledge from different free texts. Nakashole et al. [26] proposed a bootstrap method for the semi-supervised extraction of arbitrary entity relations from free-form text. Based on this algorithm, Ernst et al. [27] constructed a system called Knowlife that extracted 13 types of medical relations with an average accuracy of 93.3%. However, generalizability is not guaranteed because these methods rely on a dictionary matching method in recognition. Zheng et al. [28] treated entity recognition and relation extraction in knowledge building as a joint learning problem on the text sequence and performed end-to-end learning with a deep network, which can directly derive knowledge graph triples from text sequences. Although directly extracting semantic triples can avoid error propagation, the methods still have problems handling overlapping entities and relations. Xu [29] proposed a hybrid method to handle overlapping cases, which handles entity recognition tasks as a classification task with continuous n-gram words (segment) as input. They use the Fixed-size Ordinally Forgetting Encoding (FOFE) model to encode the contexts of a word and pass through a forward network that returns the entity type. In actual industrial practice, it is necessary to automatically extract various entities and relations expressed in an overlapping manner in a short text and to transform them into structured semantic expressions at one time. Thus, we attempted to integrate knowledge embedding methods to express the semantics of characters, and deep learning knowledge extraction models, which can tag sequential characters and multiple relations between entities by considering characters in both near and far locations.

Recently, a prominent Chinese medical KB called CMeKG [30] was developed. Both CMeKG and our KB contain tens of thousands of entities and relations. However, CMeKG mainly intends for industry or institute use, and it defines a fine-grained semantic taxonomy. In contrast, our KB follows a coarse-grained semantic taxonomy to cover a wide variety of concerns of regular medical web QA service users. Our model has two advantages: (1) construction efficiency (tens of experts built CMeKG in 2 years, whereas our model only involved two students in 5 months); (2) a KB with a looser semantic taxonomy may have better generalizability [31, 32].

#### Research on knowledge-based QA techniques

With the rapid development of KBs, QA researchers tend to adopt a knowledge graph or KB in their QA systems to improve performance. KB research has two main directions in QA systems: semantic parsing (i.e., transforming questions into intermediate logic forms for querying answers) and information retrieval (i.e., matching distributed representations of candidate answers and questions) [33, 34].

##### Knowledge-based semantic parsing

The main principle is to transform a question into a graph representation that can more accurately represent the user’s intention. Traditional methods mainly use rules or templates to match a KB subgraph, where the system searches the KB graph from known entities and relations to target answers. For example, Berant et al. [35] built a parser to construct a graph of entities and relations by matching its lexicon parsing results to a KB. However, such methods require a high labor cost, and their ability is constrained by predefined logic that may limit the coverage of the graph search. Recent works have attempted to train a parser using supervised learning. Yao et al. [36] trained an information extraction model that calculates the probability score of an entity and relation implied in a question based on the words and relations of its parsed grammatical tree. The model filters unimportant entities and selects the most likely answer for the question type. Similarly, Hu et al. [37] proposed a model to train the weight on matching entities and relations between a grammar dependency tree and knowledge subgraph. More recently, researchers attempted to encode graphs into distributed representations. Xu et al. [38] proposed the Graph-to-Seq model, which retains the semantic relations in the graph in the resulting vectors, and the vector representation achieved state-of-the-art performance. Liang et al. [39] proposed a reinforcement learning algorithm to train the encoder, which can avoid additional annotation tasks.

##### Knowledge-based information retrieval

The main principle of this method is to transfer questions and answers into embedding vectors for semantic matching. Bordes et al. [31] were the first to train a model by vectorizing the questions and matching the best answer vector. Compared to semantic parsing, vector-based methods reduce many artificial and linguistic rules. However, the choice of vectorization methods affects the expressiveness and performance. Zhang et al. [21] used a bidirectional long short-term memory (LSTM) combined with an attention mechanism to extract features of user questions for vectorization training and obtained an F1 score of 42.6 on web question sets. He [40] trained their system on the encoder-decoder framework. The system encodes the question and candidate knowledge with attention and decodes candidate knowledge into a text answer. The system performed exceptionally well on a simulated dataset with an F1 score of 91.6; however, it achieved an F1 score of only 60 on an actual dataset. Sharma et al. [41] summarized some works that used neural networks in QA and concluded that LSTM enables multi-purpose QA, that a memory network saves task information and achieves high accuracy, and that an attention mechanism helps focus on different parts of the content [20]. Researchers have also used convolutional neural networks (CNNs) for encoding and fusing different knowledge bases [42]. For complex questions, the multi-hop inference is necessary, memory networks [33] are widely used, and reinforcement learning demonstrates potential in encoding training [43].

##### Our consideration

Inspired by previous research to reduce the work and time required to build our KB-QA system, we aimed to build a QA system that integrates human-understandable knowledge and machine learning techniques, such as IBM Watson [8]. In question analysis, we had to learn a model to recognize entities and relations that could reuse the processing model for knowledge extraction from a corpus. Because our task to detect the question type is not complex, we can use simple rules in many cases. For answer retrieval, embedding vectors could be used to improve performance, and we thus adopted vector matching methods. We also found that memory- and attention-based networks can help improve precision for automatically focusing answers, avoiding enormous effort for building inference mechanisms.

## Implementation

### Data overview

The medical health data used in this study mainly consisted of two parts: EHRs from a hospital and internet medical QA data. We use the EHR data to construct the medical knowledge graph because they are high-quality expert data. The internet data contained a sufficient number of answers for ordinary users’ questions to train the QA system.

**Fig. 1:**
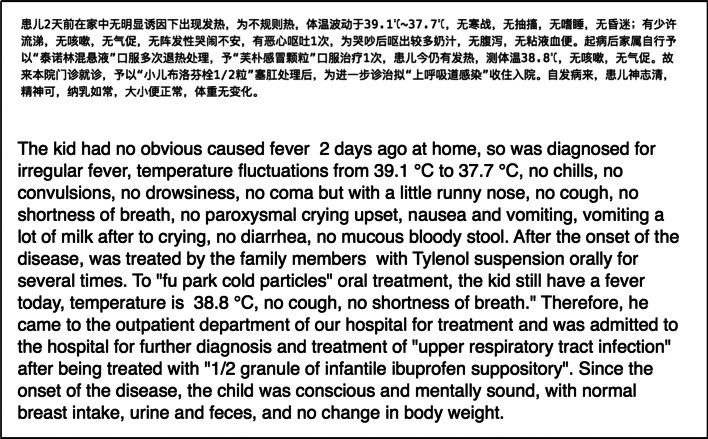
Electronic health/medical record. A child’s flu

We collected the EHR data from a hospital in Zhejiang Province and consisted of the disease histories of past and current anonymous patients. The data introduced the patient’s primary condition through an unstructured text field. The data contained many concepts of diseases, symptoms, treatments, examinations, examination results, and other related information. The dataset has 210,175 records; each record has statements about relationships between these concepts. As our estimation, there are 3–4 statements in one record, so the dataset will have about 700,000 medical statements, whose statistics are shown in Table 1. As the table shows, the records cover the central departments in a hospital, and the records from different departments vary in their main contents. We have sampled 3000 records according to their proportion, and calculated their average statement size (Column State), an average record contains about 3.3 statements. We also illustrate a record as example in Fig. 1.

**Table 1:** Statistics on EHR dataset

Departs	Records	Contents	States
Internal med	28,762	Symptom, Disease, Treat	3.5
Surgery	25,854	Symptom, Disease, Treat	2.7
Gynecology	18,833	Symptom, Disease, Treat	3.8
Pediatrics	21,981	Symptom, Disease, Treat	3.2
Infectious diseases	35,116	All	3.8
ENT	21,355	Symptom, Disease, Treat	3.0
Chinese Medicine	22,169	Symptom, Disease, Treat	4.0
Laboratory	16,011	Disease,Test, Result	3.1
Radiology	9886	Disease,Test, Result	2.3
ultrasound	10,208	Disease,Test, Result	2.5
Total	210,175	All	3.3

**Fig. 2:**
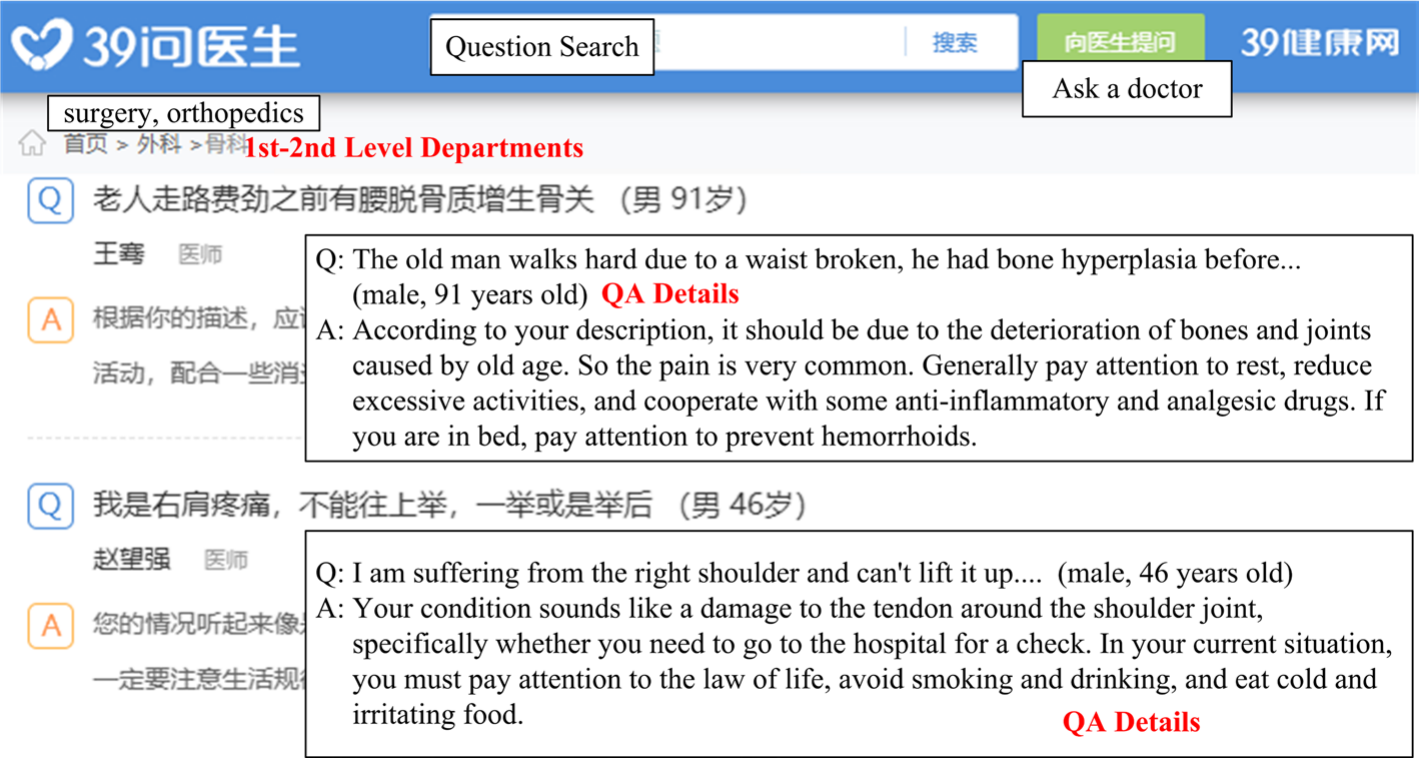
Example of question answering (QA) data crawled from the internet. Orthopedics problems

We obtained the internet QA data (QA corpus) from 39 Health Network^1^, which contained 3.02 million QA pair documents. Each data entry consisted of four parts, including relevant and secondary departments, question details, and detailed answers. Figure 2 illustrates the detailed information (in red labels) we crawl from the 39 Health Network to form the QA corpus. The statistics about the QA corpus are shown in Table 2, directly counted during crawling, from which we can see that most questions are for diseases, treatments, and tests containing a sufficient amount of QA pairs.

**Table 2:** Statistics on QA corpus

Departs	QAs	Disease	Treatment	Test
Internal med	431K	202K	213K	155K
Surgery	366K	267K	282K	275K
Gynecology	298K	241K	187K	164K
Pediatrics	281K	273K	226K	178K
Infectious diseases	351K	306K	274K	231K
ENT	255K	219K	198K	196K
Chinese Medicine	469K	443K	402K	460K
Laboratory	287K	278K	96K	261K
Radiology	118K	93K	2K	90K
Ultrasound	165K	144K	28K	121K
Total	3021K	2466K	1908K	2131K

### Method overview

**Fig. 3:**
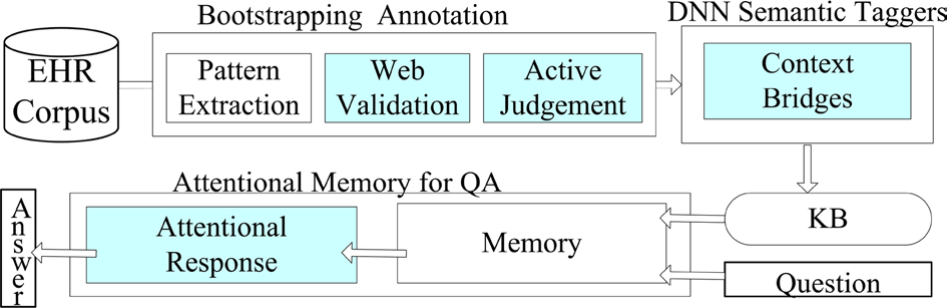
Method overview

We aimed to obtain a medical KB from many multi-source EHRs and build an intelligent medical QA system. We proposed an end-to-end method to reduce human effort, as illustrated in Fig. 3. Our method has three main building blocks: bootstrapping annotation, DNN semantic parsing, and an attention memory for QA. To integrate the blocks and improve the results, we proposed novel components and flows, which are indicated in blue in the figure.

First, we developed a bootstrapping-based method to automatically annotate semantic information (i.e., entities and relations) from the EHRs. This method iteratively mines patterns and new seeds. We used a validation mechanism that actively queries web encyclopedias or humans to improve the mining quality.

Second, we used the labeled entities and relations to train DNNs as semantic taggers that can extract new medical entities and relations by sequentially parsing an arbitrary medical text, which constitutes a medical KB. With the taggers, we can also obtain a vectorization mechanism to encode the semantics of a word or context, which is essential in knowledge construction and QA. We provided new mechanisms called **context bridges** that provide additional text context as training features for the entity and relation recognition models to improve accuracy.

Lastly, we trained a memory neural network (MemNN) to answer medical questions in the native language with the knowledge graph and internet QA corpus. We first stored the vectorized knowledge graph in the MemNN. Then, we used an attention response mechanism borrowed from computer vision research to fetch a more closely related memory to improve precision and recall.

**Fig. 4:**
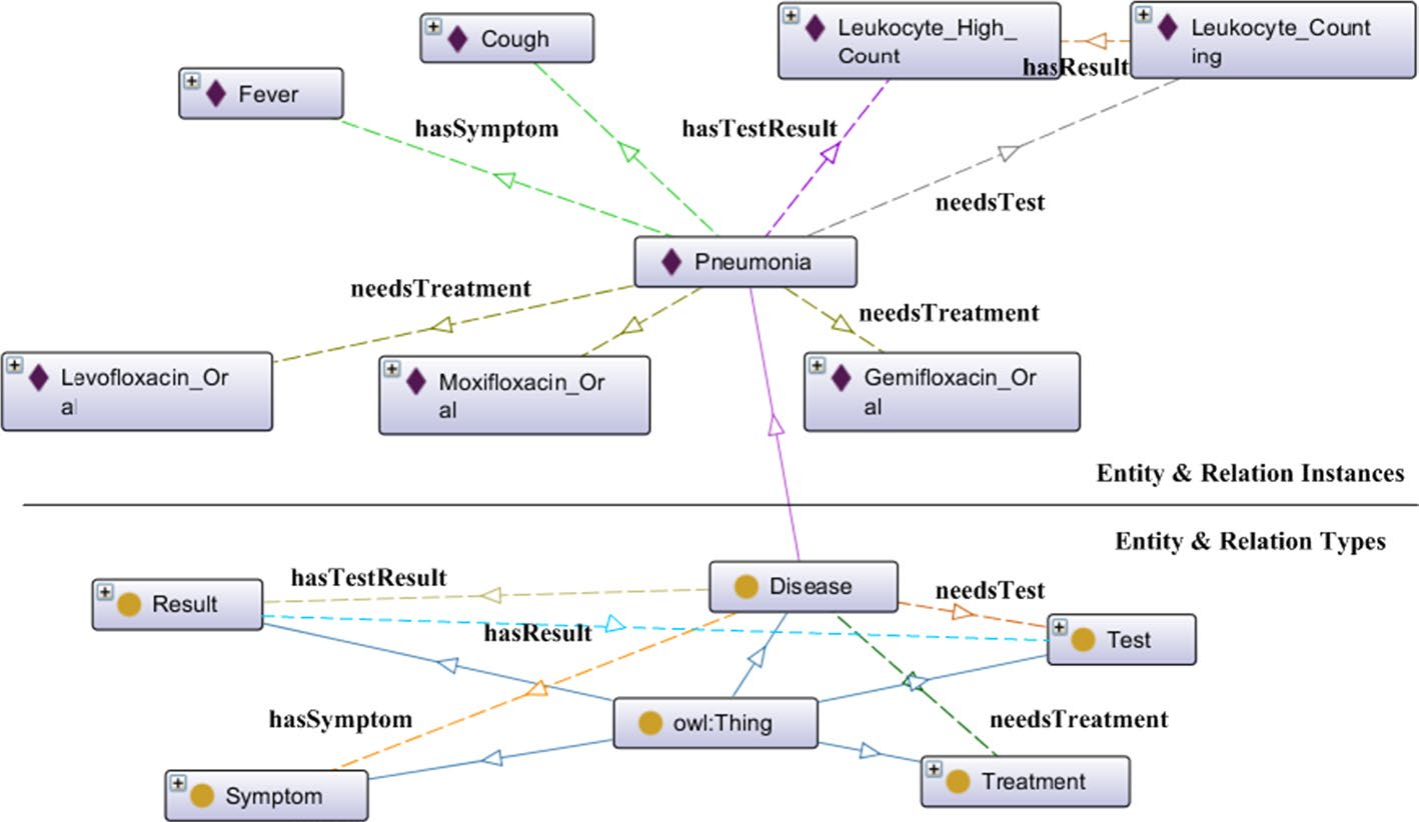
Knowledge graph ontology: model and instances. Concept of Pneumonia

### Knowledge ontology definition

In order to make a Knowledge Base, we should first define an ontology model for it.Because we aim to help ordinary users of medical websites find related information, we did not need to use a strictly scientific taxonomy, such as MetaMap or the Unified Medical Language System (UMLS) [9, 44], which are too complex for use. Instead, coarse-grained semantic categories were sufficient [45, 46]. Thus, we built a knowledge graph following the content organization of 39 Health Network, defined in OWL ontology (Fig. 4, rendered in OntoGraf). The lower part of the figure shows the ontology definition, and the upper part is an example instance of it.

**Table 3:** Relations and knowledge tuples

Type	Explanation	Subject	Object	Example KB Tuple
**hasSymptom**	a diease has a symptom	Disease	Symptom	$\langle$“pneumonia,” **hasSymptom**, “fever”$\rangle$
**needsTest**	a test helps to diagnose a disease	Disease	Test	$\langle$“pneumonia,” **needsTest**, “LeukocyteCounting”$\rangle$
**hasTestResult**	a test result indicates a diease	Disease	Result	$\langle$“pneumonia,” **hasTestResult**, “LeukocyteHighCount”$\rangle$
**needsTreatment**	a treatment cures a disease	Disease	Treatment	$\langle$“pneumonia,” **needsTreatment**, “Levofloxcin”$\rangle$
**hasResult**	a test can have a result	Test	Result	$\langle$“LeukocyteCounting”, **hasResult**, “LeukocyteHighCount”$\rangle$

The ontology is consisted of five types of entities (OWL classes) defined as follows:

1. **Disease**: represents diseases, injuries, and other health problems raised by patients, which is the core class of the ontology.
2. **Treatment**: represents treatment techniques. Since doctors have different styles in writing EHR, this class will include operations, medical treatments, drugs, and other tools, such as instruments that are used in treatment. Another reason to include treatment tools in this class is that ordinary users may not distinguish between treatment and medicine in their questions.
3. **Test (Examination)**: represents medical procedures performed to detect, diagnose, or monitor diseases, disease processes and susceptibility, or to determine the course of treatment. It also includes methods of measuring biochemical indicators and observing lesions.
4. **Symptom**: represents self-reported feelings and observable phenomena indicating diseases.
5. **Test Results**: represents quantitative measurements of indicators, qualitative results from visualization tests, and conclusions after a test.

We define five types of relations (expressed with OWL Object Properties) between the five types of entities, as shown in Table 3. Also, see the upper part in Fig. 4 as an example mined from EHR: the classes and relations have instances; disease *pneumonia* has indicating symptoms *fever* and *cough*, and it has an indicator of *high leukocyte count* (other tests and results omitted here) after a test of *leukocyte counting*; after diagnosed as pneumonia, the patient should take drugs orally (*levofloxacin*, *moxifloxacin*, *gemifloxacin*) for treatment. The relations between entity instances constitute the knowledge base stored in a Neo4j DataBase.

One more thing to mention is a Result entity like “Leukocyte_High_Count”, a semantic class representing a qualitative result. In order to have a quantitive ground, the entity will have one more Property Relation to a real-valued measurement standard. So we also defined a OWL Data Property named ’DataGrounding’, linked to a native quantitive representation of OWL language.

### Knowledge extraction

The knowledge construction process is to find instances of entities and relations according to the ontology model and fill them into a database. The process consists of three stages: **entity extraction** (extracting entities of the five types), **relation extraction** (extracting five types of relations between entities), and **knowledge fusion** (aligning entities and solving conflicting relations to form a knowledge graph).

### Entity extraction

#### Automatic training bootstrapping annotation

**Fig. 5:**
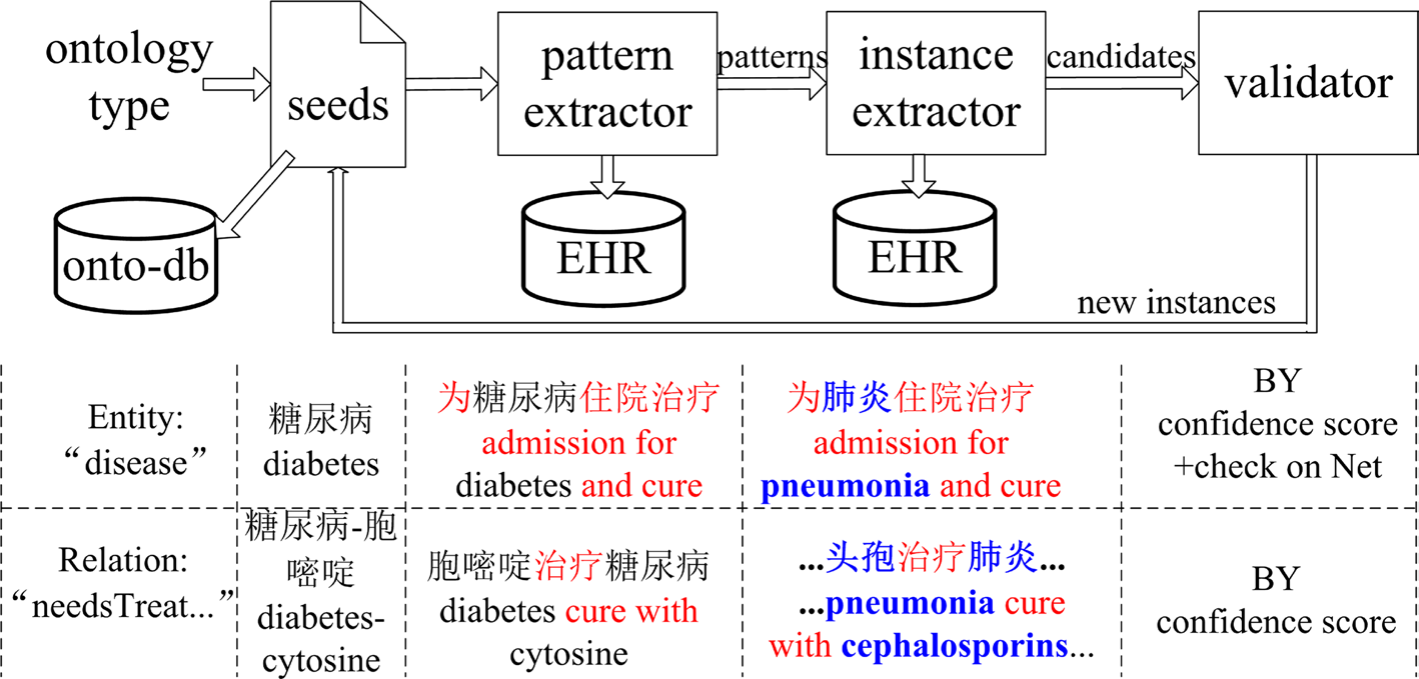
Bootstrapping text mining algorithm for EHR in Chinese

Because annotated health record data are limited, we used bootstrapping and pattern mining methods to label entities from the text. We propose a unified bootstrapping algorithm for both entity and relation extraction (illustrated in Fig. 5).

##### Seed annotation

Given a specific entity type, bootstrapping starts with a set of seed words, which are manually labeled entities (seeds are presented in Additional file 1: Appendix 1). In Fig. 5, given entity type Disease, an expert provides a seed word *diabetes*.

##### Pattern extraction

We use Chinese characters surrounding a seed word as an indicator to determine whether it is an entity of the target type. The surrounding characters are called a **pattern** to extract an entity for a type (like the red text surrounding the seed word *diabetes* in Fig. 5).

In detail, given a seed, the algorithm searches for patterns in the EHR text. When detecting a seed in a sentence, the algorithm extracts 1 to 3 characters to the left (right) of it, which forms a set of its **left (right) contexts**. A combination of a left context and a right context is a **candidate pattern**, and an independent left or right context is also a candidate pattern. For example, in the sentence “treatment admission for diabetes and cure with cytosine,“ we detected the disease seed word “diabetes” and a treatment seed word “cytosine,” and thus obtained a candidate pattern “admission for $\langle DIS\rangle$ and” for disease, and a candidate pattern “cure with $\langle TRE\rangle$ [END]” for treatment extraction, where “$\langle DIS\rangle$” and “$\langle TRE\rangle$” are placeholders of *disease* and *treatment*.

To decrease noise, it is necessary to discard unqualified candidates for patterns according to certain criteria. For pattern evaluation, we calculated the **support value** [47]^2^ and **confidence value** [48]^3^, and then the **reliability score** of each candidate pattern. To calculate the support and confidence values, we first introduced two sets: (1) a word-with-pattern set *Pt*(*s*) was defined as a word set extracted with a pattern *s*, which had an intersection with the seed set, and (2) a word-with-type set *Tp*(*c*) was defined as the entity set with type *c*. Then, the values and scores were calculated via (1)–(3).

1 $$\begin{aligned} Support_s= \frac{|Pt(s) \cap seeds|}{|Pt(s)|} \end{aligned}$$

2 $$\begin{aligned} Confi_{s,c}= \frac{|Pt(s) \cap seeds \cap Tp(c)|}{|Pt(s) \cap seeds|} \end{aligned}$$

3 $$\begin{aligned} Score_s&= w\cdot Support_s+(1-w) \\&\times max_c(Confi_{s,c}) \end{aligned}$$

A pattern with a score higher than a certain threshold (0.7) was validated, while others were called non-validated patterns, and the strings extracted with the valid pattern were considered **candidate entities**.

##### Instance (entity word) extraction

Bootstrapping is an algorithm that alternatively and iteratively extracts new patterns and entities with the previous round’s result as input of the following round (like the blue word *pneumonia*, surrounded by red text pattern) in Fig. 5.

After obtaining candidate patterns, they were then used to mine additional words from the EHR. The algorithm uses the pattern to match EHR text. For a text, the left and right contexts are marked, and the text between them is a candidate for a new seed if it satisfies certain conditions, such as having a particular length or being in one sentence. For example, if we search for the pattern “admission for $\langle DIS\rangle$ and” and find the text “...admission for pneumonia,” then “pneumonia” is a candidate for a new instance of disease.

##### Entity validation

To validate the candidate entities, we searched for them in web encyclopedias (i.e., Baidu Baike^4^ and 39 Health Network). If the candidate entity appeared as an entry of the encyclopedia, it was considered a valid entity and placed into the seed entity set (called **new seeds**). If a candidate entity did not appear as an encyclopedia entry, it was sent to a human judge for manual validation.

To improve the recall, we extracted entity candidates from non-validated patterns with a score higher than 0.3 were also verified using an internet search engine. Let *H* represent the set of search results that contained the word in the top K results. Human judges determine whether an entity with a score ($Score_c=\frac{|H|}{K}$) more significant than the threshold is an entity. For example, due to typos in EHRs, patterns like “recognized as” are misused as “diagnosed as” (typical in Chinese EHRs). Such patterns have low scores but can also obtain correct disease names, such as “congenital heart disease.” When we used a search engine to search for this phrase, it was the only entry from 39 Health Network (without other results); thus, we recognized it as an entity.

##### Work in loops

After validating candidate entities, we put them into the seed list as new seeds. The new seeds further generate additional **patterns**. The mining procedure for new patterns and new seeds repeats iteratively until the number of seed words does not increase (i.e., there were any new seeds). Finally, all the seed words were labeled as entities and put into the knowledge base.

#### Conditional Random Field + Bidirectional LSTM (CRF+Bi-LSTM) model for entity recognition

**Fig. 6:**
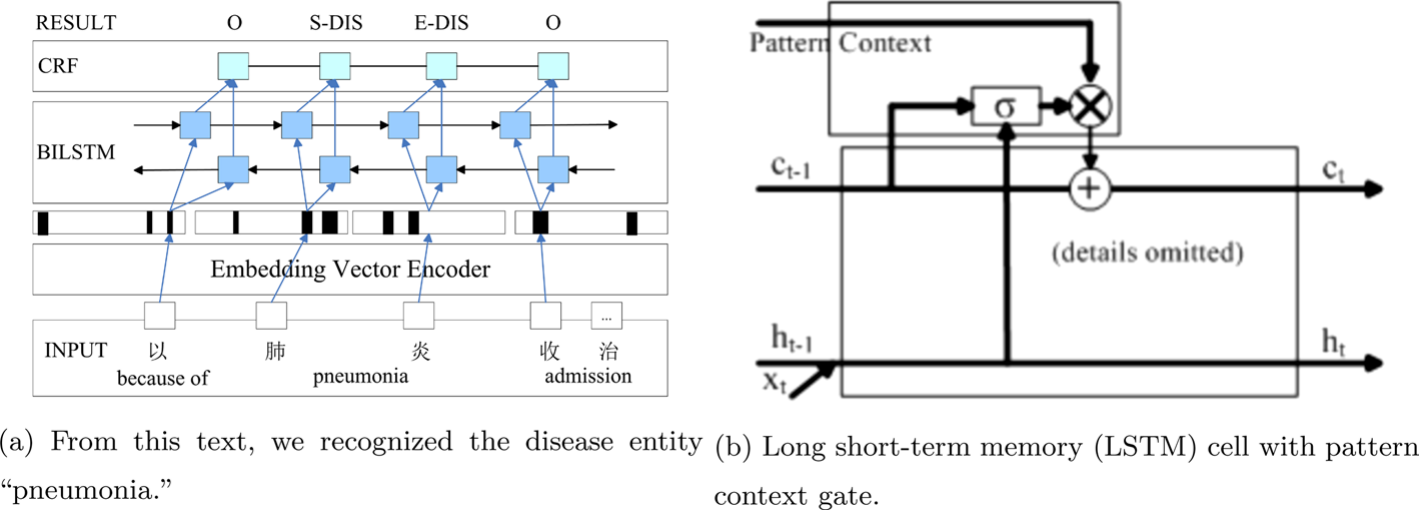
Bi-LSTM+CRF structure

We trained a learning model for medical entity recognition to be a more automatic tool for labeling entities from text. Our proposed method is a hybrid structure combining an embedding encoder for word vectorization, a bidirectional LSTM (Bi-LSTM) model that vectorizes text sequences, and a conditional random field (CRF) model that recognizes entities from the text. We first extracted features (which were individual Chinese characters, not segmented strings, in contrast to [1]) of the text flow using the Bi-LSTM layer, and then used the features in the CRF layer to obtain global label prediction on both the tokenization of the entity and its type, as illustrated in Fig. 6a.

Notably, there are two differences between our model and the original Bi-LSTM+CRF model proposed in [49].

1. Our model employs an embedding vectorizer for input preprocessing trained together with the Bi-LSTM and CRF. There are two options for embedding: training with the model (co-training) and pre-training with an embedding tool, such as Google word2vec. As demonstrated in the Results and Discussion section, the performance of co-training is better than that of pre-training.
2. We slightly modified the LSTM cell. Because an entity may depend more on near text contexts (surrounding character patterns, as in bootstrapping), we added a context bridge in the LSTM cell, as illustrated in Fig. 6b. When tagging a character, its surrounding characters are used to find a list of similar patterns extracted during bootstrapping. A pattern is transferred into a vector concatenating all of its character vectors (we used co-trained vectors from the entity extraction module and constrained the length of a pattern to eight characters by selecting the leftmost and rightmost characters or padding patterns shorter than eight characters). The pattern vectors then became another input for entity recognition through the bridge, like the gate model in [45]. In the extended LSTM cell, the cell status is modified as $c_t=c_t^{orig}+p_t*pt$, where $c_t^{orig}$ is the original calculation function of the cell state, and *pt* is the pattern vector. The probability that the additional input pattern take effects $p_t$ is calculated as $p_t=\sigma (W_{pi}[H_{t-1},x_t]+W_{pc}c_{t-1}+b_p)$, where $H_{t-1}$ and $x_t$ are the hidden state of the last time and current input, respectively. $W_{pi}$, $W_{pc}$, and $b_p$ represent trained parameters.

In our model, the CRF layer represents a matrix *A*, which represents the transition score between entity type labels on successive characters (“*S-DIS*” and “*E-DIS*” represent a starting character and an ending of a disease word). The output from Bi-LSTM into CRF is a prediction matrix *P* representing the prediction probability of labeling a character with an entity type label. The total prediction score that labels the word sequence *x* with the label sequence *y* is presented in (4), where *t* is the position and *y* is the label.

4 $$\begin{aligned} score(x,y,{\widetilde{\theta }})=\sum _{t=1}^{T}(A_{y_{t-1},y_t}+P_{t,y_t}) \end{aligned}$$

We divided the data into batches during the model training process and iteratively trained the model. The training process followed classic forward and backward propagation for each batch (50 labeled sentences) in each iteration.

### Relation extraction

Relation extraction determines the internal relations between entities, such as dependency, taxonomy, and cause, which are the core link of semantic information and the basis of the knowledge graph. Relations between entities form a semantic graph, which is the key to finding an answer to a user’s question.

Due to the lack of annotated data, we used a process similar to entity extraction that involved first bootstrapping to create training data and then training a supervised model. We used a CNN for learning.

#### Bootstrapping annotation

This study first manually prepared seed relations for different semantic types (listed in Additional file 1: Appendix 2). Then, we mined patterns for them and extracted new relations (i.e., new seeds) with validated patterns. This procedure iteratively extracted new patterns and relations until nothing else was extracted; see Fig. 5 for the algorithm and example.

The differences of relation extraction from entity extraction are as follows (shown in the lower row in Fig. 5): (1) The seed is a tuple specifying two entities and their relation, such as $\langle$“diabetes,” **needsTreatment**, “cytosine”$\rangle$. (2) The extracted patterns are called a context string, a string between entity pairs and is at most one word left of the first entity and at most one word right of the second entity in the same clause. For example, in the sentence “admission for diabetes diagnosis and cytosine treatment,” the context string of the entities “diabetes“ and “cytosine“ is “admission for _ cure with _ .“ Then, we segment the context string into a word sequence with a dictionary (including extracted entities) and the bi-directional matching algorithm, filter the words with a stop list and term frequency-inverse document frequency value in the EHR, and enumerate the substrings of the word sequence. Then, the string and its substrings are considered candidate patterns of the **needsTreatment** relation. Note that a pattern string can also link entities across multiple sentences. Examples of the string pattern extraction process are in Additional file 1: Appendix 3. The definition of pattern reliability metrics is nearly the same in (1)–(3); however, we redefined the components of the formulas: (1) a pair-with-pattern set *Pt*(*s*) is defined as a set of entity pairs extracted with a pattern *s*, which has an intersection with the seed set of the relation type; and (2) a pair-with-type set *Tp*(*c*) is defined as the extracted pair set with the relation type *c*. The selected patterns are called **string patterns**.

#### Training a CNN model to recognize relations from medical text

**Fig. 7:**
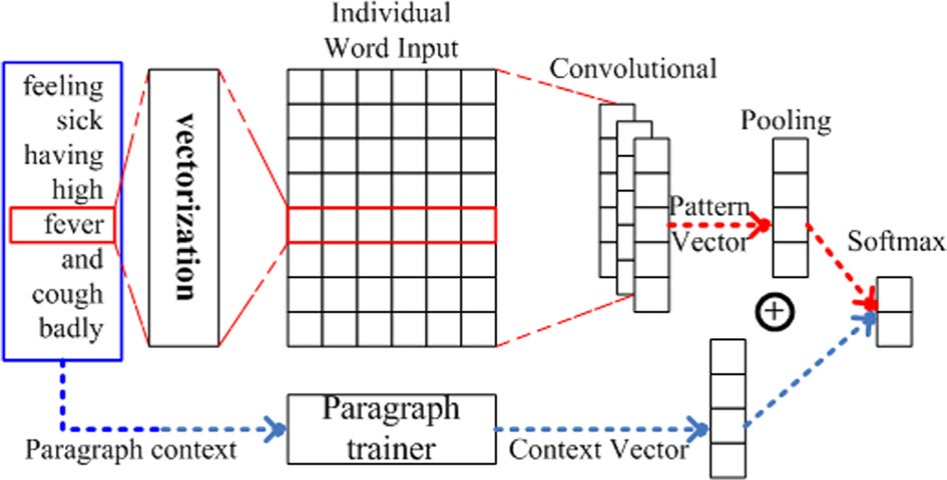
Convolutional neural network (CNN) relational extraction model structure

To automatically extract relations from text, we trained a model. Because a text can be regarded as a one-dimensional image with dense information, a CNN is also effective in coarse-grained natural language processing tasks, such as relation extraction. A CNN can automatically detect deep syntactic features and sensitively extract unique syntactic features concentrated in a local part of the corpus. Considering that the length of a natural language is not as fixed as that of an image, our relational extraction algorithm adopts the CNN model with a sliding window. The CNN relation extraction model is presented in Fig. 7. In training, the model takes the string patterns defined above as input, while in testing, the model takes substrings of the context string of entities as input. The window (shown in the blue rectangle) slides on the input after the word segmentation and vectorization.

***Single-Word Features*** Let $x_i\in R^{k}$ be the K-dimensional vectorized representation of the i-th word (entity or non-entity word) in the window. Then, the window with length n (those that are insufficient must be completed) can be expressed as $x_{1:n}=x_1\oplus x_2\cdot \cdot \cdot \oplus x_n$, where $\oplus$ represents concatenation. Let $w\in R^{hk}$ be the convolution kernel with a size of $h\times k$. The feature *c* of the convolution operation can be expressed as (5) and (6) as follows:

5 $$\begin{aligned} c_i= f(w\cdot x_{i:i+h-1}+b)\,\,\,\# \end{aligned}$$

6 $$\begin{aligned} c= [c_1,c_2,ldots c_{n-h+1}] \end{aligned}$$

Then, with maximum pooling, the feature graph *c* is changed to ${\widehat{c}}=max\{c\}$, and the entire feature graph is represented as the feature with the largest value with a decreased calculation cost.

##### Pattern context vector (context bridge)

The whole paragraph can provide additional information in mining relations, so we call it a **pattern context**. We follow the pattern vectorization proposed by Le et al. [50], which is a process that transfers a paragraph into a vector using a procedure similar to word embedding algorithms. As illustrated in Fig. 7, an additional vector for the paragraph context is learned with the backpropagation algorithm while learning the embedding vectors of all words in the paragraph. We directly applied the algorithm on the entire corpus; thus, each paragraph had a vector embedding from its bag-of-word representation. For a new paragraph, we learned its vector by predicting the following word in the paragraph while the word embedding remained fixed.

##### Classification

After obtaining all pooled single-word feature graphs from all convolution kernels and obtaining the paragraph vector, we concatenate ($\oplus$) the feature vector from single-word features and the pattern context vector. Then, classification is performed with a fully connected softmax layer. The definition of the model score is presented in (7), and the optimal model solution is obtained by maximizing this function, where $y^{(i)}$ is the relation class of the $i-th$ training instance and $sp^{(i)}$ is its input (string pattern).

7 $$\begin{aligned} score_{\theta }=\sum _{i=1}^{T}logp(y^{(i)}|(sp^{(i)},\theta ) \end{aligned}$$

During the implementation, because maximum pooling discards certain unimportant features, the CNN cannot use the co-training methods that train the word vector matrix and model together. Therefore, when addressing native language tasks, a CNN often adopts the method of pre-training the word vector matrix and fine-tuning during model training to perform word vectorization.

### Knowledge fusion

The entity and relation extraction results often contain redundant and erroneous information, and the extracted data lacks a hierarchical structure. Thus, it is necessary to perform knowledge fusion for cleanup and integration. At the same time, it is also necessary to quantify the credibility of the knowledge mining results to decide whether to add an entity or relation to the final knowledge graph to ensure its quality. In our method, knowledge fusion has two main phases: entity alignment and consistency analysis.

#### Entity alignment

Entity alignment [21] involves linking a newly extracted entity to an entity with the same meaning (if it exists) in the knowledge graph by calculating the similarity. Generally, it measures the similarity between their semantic vectors. We constructed a vectorization model to transform entities into multi-dimensional vectors; (the method is almost the same in Fig. 6.a, and the only difference is that we first tokenize the entities as a word and use the model to learn its type, after training, we use the embedding vector encoder to vectorize the entities. Then, the cosine similarity between vectors represented the similarity between entities. In this study, we compared word vectorization algorithms: we call our method the co-training method and the Google word2vec algorithm (pre-training). We selected the co-training method used in the named entity recognition model according to the performance and effectiveness.

For example, in the entity extraction results, the similarity between the two entities “fever” and “irregular heat” was very high, reaching 0.97. Therefore, we aligned the two entities as one entity with the name “fever,” which appeared the most significant number of times and was thus used as the entity name. Then, we set an empirical threshold of 0.7 to merge two entities, and a human validated the entity pair with similarity values between 0.3 and 0.7.

#### Consistency analysis

Consistency analysis refers to solving semantic conflicts (e.g., conflicting relations between the same entity pair due to different contexts) to avoid duplication in the knowledge graph. There are three main methods of consistency analysis. This study used supportability-based and artificially-based methods to resolve conflicts. We calculate supportability from the frequency of each entity or relation is validated and the frequency it appears in the text. The artificially-based method manually resolves the conflicts that cannot be solved automatically; trade-offs can be performed.

For example, the relation extraction results (in tuples) $\langle$“Respiratory infection,” disease-treatment, “Pediatrics Ibuprofen Suppositories”$\rangle$ appeared 74 times in total, while the entity “Respiratory infection” appeared 379 times; thus, the confidence of the relation was 74/379 = 0.195. The phrase $\rangle$“Respiratory infection,” disease-treatment, “antibiotic treatment”$\rangle$ had a confidence of 0.71; thus, the relation was retained. Empirically, we set the threshold value to 0.7. If the confidence was above this value, the relation was accepted. If the value was lower than 0.7 but higher than 0.3, a human judged whether to keep the relation.

### Memory-neural-network-based QA model

There are three main methods of using a KB in a QA system. First, we can match the question syntactic tree to a similar subgraph of the KB. Second, we can extract entities from the question and find related entities and relations in the KB as answers. Third, we can learn a model that understands the relationship between vectorized questions and knowledge. The third method is used to reduce the cost. Specifically, we combine an attention mechanism and memory neural network, state-of-the-art deep learning mechanisms [20], to build a unified architecture to match questions and memories to generate answers.

**Fig. 8:**
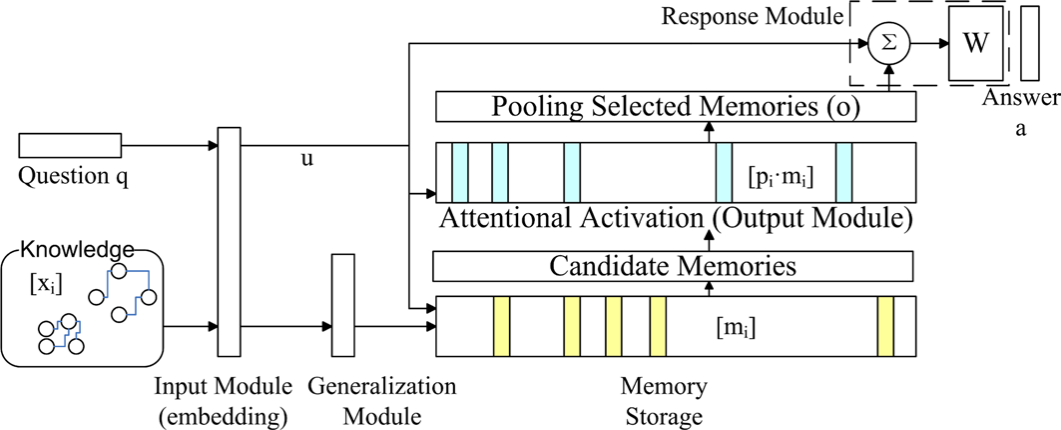
Memory neural network structure for question answering (QA) model

The core of our QA system is a memory network (MemNN) [51]. In the original MemNN, an external memory mechanism is introduced into the traditional neural network structure. Compared to the memory in LSTM, the memory in MemNN is explicit, large-scale, and long-term; it can memorize the KB without information loss when compressed into a low-dimensional space, which improves the answer accuracy. With external memory, we can also avoid retraining changes of the KB. Its updating and deletion are managed by a utility evaluation mechanism called generalization (details are presented in the Memory Management section). We believe that by memorizing the KB in the memory, the neural network can refer to the KB when it fetches potential answers to a question. We propose a new MemNN structure to train medical QA end-to-end; thus, we extended the MemNN by replacing question analysis and memory extraction with the matching of embedding vectors. Our MemNN framework is presented in Fig. 8. In general there are four main processes. Firstly, the question and knowledge will be embedding with Input Module (i.e. transfer the question *q* to embedding *u*, and knowledge $x_i$ to memory $m_i$). The Generalization Module will control whether to adopt or to drop a memory $m_i$ in Memory Storage. When answering a question, use input question *u* to match a proper memory *o* with attentional activation mechanism in Output Module. Last, to generate answer *a* with question *u* and memory *o*.

#### Input processing

There are two inputs for the MemNN: user questions (*q*) and medical EHR records $[x_i]$. Our system first translates the input into an internal representation *u* and $[m_i]$ correspondingly to improve their semantic expression ability. The internal representation consists of a vector from the embedding mechanism and the tuple representations of the extracted entities and relations.

**Fig. 9:**
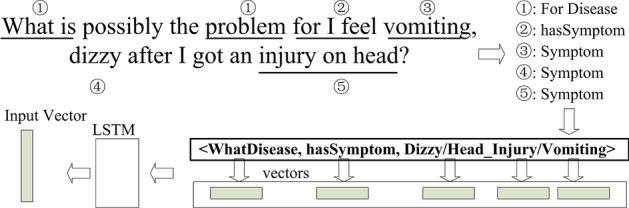
Input processing explanation

In detail, the whole process is shown in Fig. 9. First, the input is passed to the extraction methods in the last section to get entities and relations. Secondly, for efficient memory search and output, relations are combined into tuples with the same subject and entity relations, and the object is a set of multiple entities. For example, a disease may have multiple symptoms. When reading the memory, it should output the triple $\langle$*disease*, **hasSymptom**, *{symptom 1, symptom 2, symptom 3... }*$\rangle$, not multiple tuples. Moreover, the tuples are sorted with alphabet order according to their subject and object. Then, the tuples are transferred into vectors. Entities are converted into vectors with their words (using the co-train method for vectorization in the entity alignment model). The relation is expressed with a one-hot vector of its enumerative type (1 for hasSymptom, 2 for needsTest, etc.). Finally, an LSTM embedding model is trained for transforming the sub-vectors into a final vectorization.

For a question, the input processing has a certain step to detect question words that capture the apparent characteristics of the question. For example, if “what disease” is included in the search request, the user’s search intention corresponds to the disease entity, while “how to” and “how to treat” correspond to treatment entities. In addition, “symptoms” and “manifestations” correspond to the entity of symptoms and examination results. Extracted question words are treated as a placeholder of future answer entities.

#### Memory management

The memory storage module is responsible for managing the stored knowledge memories. Each piece of memory is a distributed representation of a relation tuple denoted $\mathbf {m_i}$ transformed from $\mathbf {x_i}$. For $\mathbf {m_i}$ we store both its semantic tuple and the vectorized representation in our storage. The memory module is responsible for fetching, inserting, modifying, and deleting a memory piece. Because the size of the fused KB is small (as illustrated in Table 12), one server can store it in its RAM when scaling out. We create an index for the tuples with their entities and relation type. Intuitively, the index size scales linearly with the number of tuples for a tuple includes a certain number of entities. So the searching time of memory for a question tuple is *O*(*nlogn*), in a sorted memory.

The generalization module is responsible for controlling memory reading and writing according to the user input. In original MemNN, one can build the control logic with different designs and techniques on demand.

**Table 4:** Memory management examples

Input	Input type	Memory $m_i$	Memory features	$v_n$	$v_i$	Operations
What disease has symptom: dizzy, vomiting, head Injury	question	concussion hasSymptom dizzy/vomiting	input Similarity: 0.76, used times: 0.017, entities: 3,type: 0001	0	0.83	use memory to answer question
Concussion has symptom: dizzy, vomiting, head injury	knowledge	concussion hasSymptom dizzy/vomiting	input similarity: 0.76, used times: 0.017, entities: 3,type: 0001	0.92	0.83	use input to replace old memory
000000	command for clean	flu needsTest bodyTemperature	input similarity: 0, used times: 0.00, entities: 2,type: 0100	0	0.03	useless memory, forget it

We train a multi-layered perceptron (MLP) that takes the new input *I*(*X*) and a memory piece $m_i$ to determine the utility values of the memories $v_n$ and $v_i$, then the update operations on them $o_n$ and $o_i$ are decided with rules accordingly. The module function can be written as $\mathbf {v_n, v_i, o_n, o_i = G(m_n, m_i, M)}$. This module is trained for three decision tasks: determining whether to store the input as a new memory, whether to forget a memory piece to avoid conflict, and whether to use a memory piece to answer a question (see Table 4 for working examples with memory features for judgment, in the first row, the similarity between input and memory is enough, so the memory is a candidate to be used for answering; in the second row, two knowledge are similar, so consider to replace according to predefined rules; in the third row, when cleaning the memory, the seldom-used ones are considered to be cleaned).

#### Attention response

The Output Module is responsible for selecting the most relevant knowledge in the candidate memories according to the internal representation of the question *u*. Unlike the generalization module, which selects all possible candidate memories above a line of $v_i$ to improve the recall, the output module selects only the most probable memories (Top K) with an attention-based method. The module does not use a simple selection method to compare the embedding vector of the question and memory vector and select the memory with the highest similarity as the output. Instead, using semantics, it assigns different attention weights to parts of the tuple and calculates the probability (attention score) to use the memory.

The attention mechanism follows the region affinity method applied in computer vision (CV) research [52]. Consider matrices of a question a memory (*u* and *m*), where each column of *u* or *m* ($u^i$ and every $m^j$) is a vectorized entity or relation in the matrices called a *patch*. The similarity between two patches is defined as $s_{i,j}=\left\langle \frac{u^i}{\left\| u^i \right\| _2},\frac{m^j}{\left\| m^j \right\| _2} \right\rangle$, and the attention of $u^i$ on $m^j$ is calculated as $\alpha _{i,j}=\frac{exp(s_{i,j})}{\sum exp(s_{i,j})}$. The final attention value is trained with a multi-layered perceptron as $Att=W*[aver(\alpha _{i,j}),\sum \alpha _{i,j},\max \alpha _{i,j}]$. The memory with the most significant attention score is selected. We use this attention mechanism because it considers both local and global similarities. In the figure, each patch from the question tried to match a candidate memory; after we calculated the average, summation, and maximum attention from all $\alpha _{i,j}$, we got the attention score and selected the highest memory to answer the question. An example is shown in Fig. 10.1, where the question *u* compares its columns to different memories and gets column-by-column attention $\alpha$ to calculate the final matching scores and only one memory with the highest score is used in answering the question.

The Response Module generates answers in natural language according to the question internal representation (*u*) and the corresponding memory fetched by the output module $m_o$. It is equivalent to the decoding part in every QA model: some works use question words to filter pieces from $m_o$ to find the most frequent occurred entity with the target type (like a disease) as the answer; others use an end-to-end solution like seq2seq to generate an answer sentence. A recurrent neural network is trained to predict an entity with target type as the answer, and we use a predefined text frame to generate an answer sentence. An example is shown in Fig. 10.2, for the question *u*, we put its question word (*what disease*), a memory from the Output Module ($m_1$) and *u* itself to decode an entity *Concussion* and the question type *Disease* with the trained RNN decoder, and then generate the final answer.

**Fig. 10:**
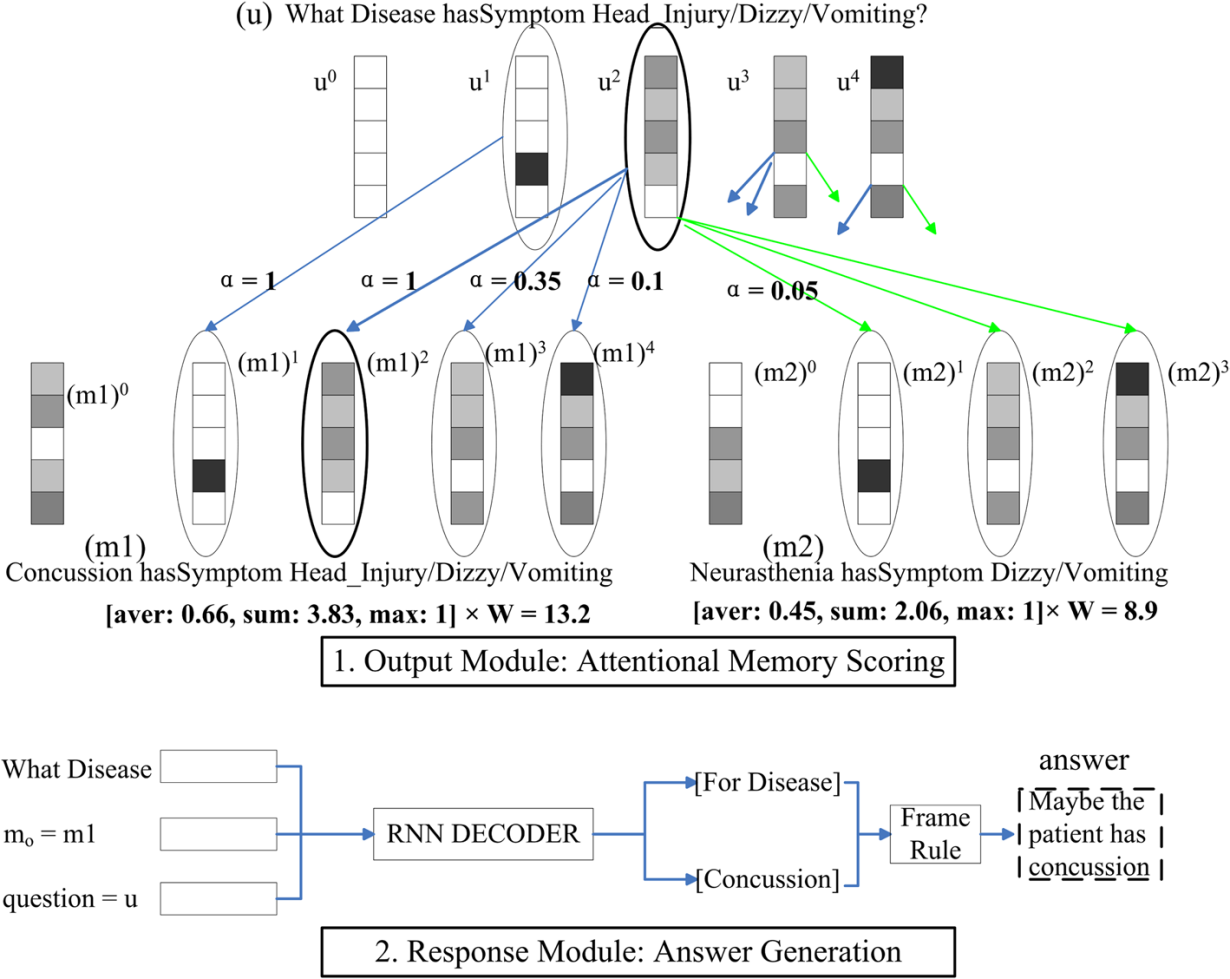
Output and response

When the question is ${\mathbf {u}}$ and the memory is $\mathbf {m_o}$, the answer is ${\mathbf {a}}$. The loss function to be optimized is defined as Formula (8), where $\gamma$ is the similarity threshold for selecting responses, ${\bar{f}}$ is an incorrect memory, ${\bar{a}}$ is an incorrect answer, $s_o$ is the similarity between the memory and input, and $s_R$ is the similarity between the response and joint vector of the input and memory.

8 $$\begin{aligned} loss= & {} \sum _{{\bar{f}}\ne m_o}max(0,\gamma -s_o(u,m_o)+s_o(u,{\bar{f}})) \\&+\sum _{{\bar{a}}\ne a}max(0,\gamma -s_R([x,m_o],a])+s_R([u,m_o],{\bar{a}}]))\# \end{aligned}$$

#### QA training

According to the MemNN structure, the QA system model construction process generally has two parts:

1. Training the output and response networks with question-and-answer pairs of the internet QA corpus. After converting EHR records into vectors and storing the KB in memory, we train the attention network in the output module to identify the most related knowledge. We train the response module to generate natural language answers according to the selected memory and input question. To avoid traversing all memory, it is often appropriate to first match all entities in the problem with all entities in the memory module to determine the search scope and then calculate the similarity to identify the memory. Given a QA pair as a positive example, a negative sample of an answer and memory is sampled from the corpus or memory base, which does not contain entities in the question and answer. The network loss function is presented in (8).
2. Training the generalization module. After training other modules, we train the generalization module, which determines the utility value of knowledge tuples for different inputs. We judge each memory $m_i$ in the memory module with all QA pairs for its value. Then, the same network training steps of 1) are executed again. During this procedure, the generalization module is supervised with Formula (8) that when a memory helps conduct correct answer, its value is 1, and usage counting adds 1. The gradients are back-propagated to the utility nodes of the generalization module.

After each module is fully trained, we obtain a complete QA system. It not only provides answers to native language questions of users by retrieving knowledge in memory but also modifies the knowledge at any time.

Our study placed the knowledge triples extracted from our EHRs into memory and later trained the network with our internet QA data. Then, if the system received a new question, such as in Fig. 2, it first extracted entities such as “hyperplasia,” “bone pains,” “waist broke,” and a pattern such as “what is wrong I feel...” that implies that this was for disease. Later, the system identified the most closely related memory, such as $\langle$“bone and joint deterioration, ”hasSymptom, “bone pains”$\rangle$. Then, the system generated the answer according to the memory, such as “it seems to be bone and joint deterioration....”

## Results and discussion

In this section, we evaluate the performance of knowledge extraction, which is mainly measured by extraction accuracy. Then, we evaluate the quality of single-fact QA tasks in which we compare different models. The evaluations are based on our five biomedical semantic categories.

### Entity extraction performance

#### Data annotation quality

**Fig. 11:**
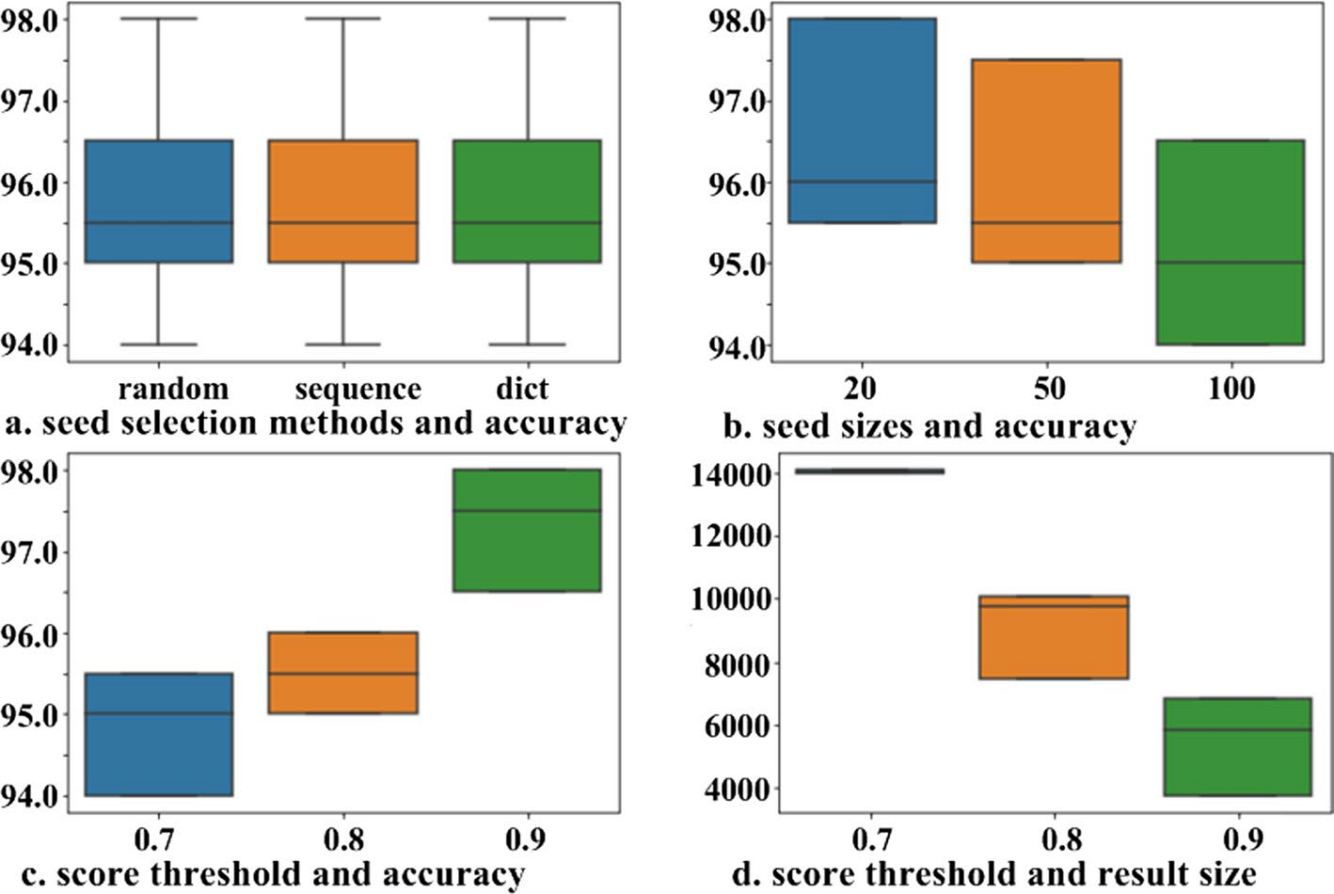
Results of mining disease entity with different seed word selection methods and score thresholds

The method of selecting bootstrapping hyperparameters affects the annotation results. Thus, we first optimized the hyperparameters (i.e., seed words and score threshold). In bootstrapping, seed words are often selected from the text in one of three ways: randomly selected from a manually labeled set, sequentially selected, and selected from a dictionary. To optimize the performance, we attempted various combinations of different seed selection methods with different thresholds and randomly investigated 200 extracted entities on a short mining trail to observe the precision. According to Fig. 11, (a) the seed selection methods did not lead to a large difference in extraction accuracy; (b), however, the initial seed size did^5^. (c) Although setting the threshold to 0.7 leads to 2% lower accuracy, (d) it can greatly improve the coverage^6^. Thus, we set the initial seed set to be 20 words, and set 0.7 as the threshold in the system.

**Fig. 12:**
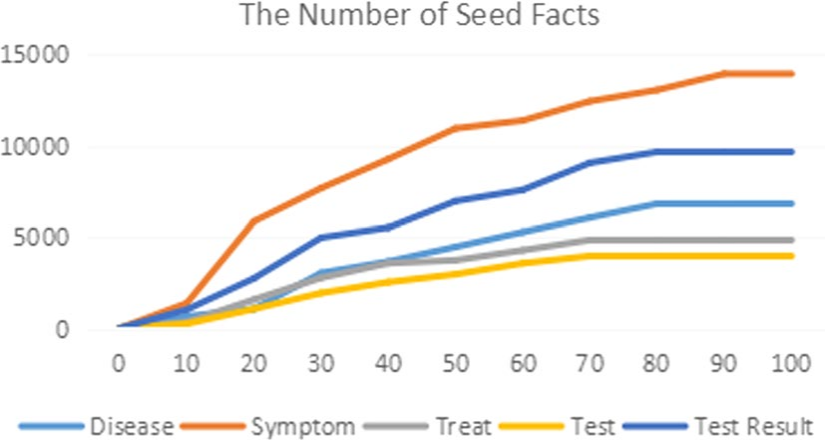
Increase curve for each solid seed entity type per iteration

Figure 12 presents a graph of the increasing trend of mined entities of each type changing with the number of iterations, in which the seed words converged after 100 iterations.

#### Performance of trained model

After we obtained the annotated data, we randomly divided them into training, validation, and test sets at a 3:1:1 ratio. Training used the validation set to judge when to stop the learning, and the evaluation method checked a randomly selected set (200 entities evaluated by a human) from the test set (presented in Additional file 1: Appendix 4) to calculate the F1 score. Then, we trained the entity classification model and compared the different models (CRF, Bi-LSTM, and combined model) and different word vectorization methods (pre-training and co-training) with the F1 score as the measurement. The hyperparameters are provided in Table 5, and the results are presented in Table 6. The method combining CRF and Bi-LSTM, together with co-training word vectorization, achieved the highest performance. It should be noted that the evaluation was based on our coarse-grained semantic categories.

**Table 5:** Hyperparameter settings for entity extraction

Hyperparameter	Setting
Batch size	16
Embedding dimension	512
LSTM layer	1
LSTM hidden nodes	200
CRF input dimension	200

**Table 6:** Final model results for entity extraction

Model	Word vectorization	F1 Score
CRF	Pre-train	0.8132
Bi-LSTM	Pre-train	0.8341
Bi-LSTM+CRF	Pre-train	0.8563
Bi-LSTM	Co-train	0.8427
Bi-LSTM+CRF (w/o context bridge)	Co-train	0.8590
Bi-LSTM+CRF	Co-train	0.8894

### Relation extraction performance

#### Data annotation quality

**Fig. 13:**
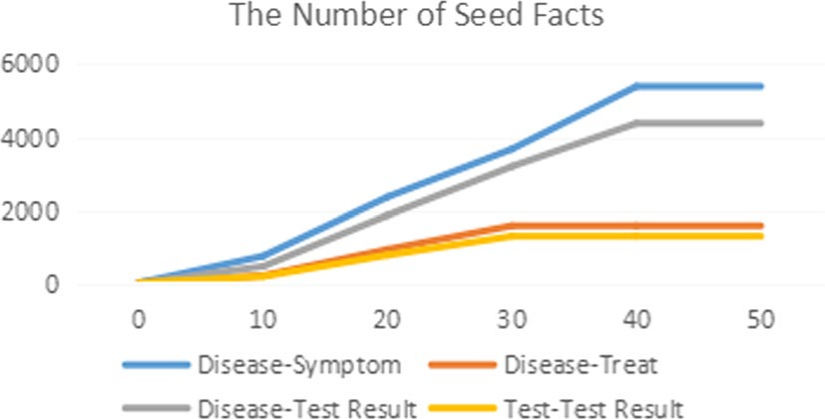
Increase curve for each solid seed relation type per iteration

When annotating relations in the EHR using the bootstrapping method, we also evaluated different combinations of hyperparameters (seed size and score threshold) and selected an initial seed size of 20 and a score threshold of 0.7 to balance the result amount and accuracy. The bootstrapping results are presented in Fig. 13.

#### Performance of trained model

After obtaining the annotated training data, we also randomly split them into training, validation, and test sets at a 3:1:1 ratio for model training and evaluation. The training and evaluation procedure was the same with entity extraction (200 randomly selected test cases were verified by a human). The verified test cases are provided in Additional file 1: Appendix 5. We selected two optimized parameters: the convolution window (i.e., number of words) of the CNN and the dimensions for word vectorization. During the evaluation, we also used the F1 score on the validation set as the measurement. As the results indicate in Table 7, we set 100 as the dimension of the word vector, and 5 as the convolution window size. The CNN parameters included one convolutional layer that contained 16 convolutional kernels, each of which had a width of half of the input sequence width and a height of half of the word vector dimension. Following the convolutional layer was a fully connected layer with 128 nodes.

**Table 7:** Parameter selection results for relation extraction

CNN Window	Word Vec Dim	F1 Score
3	50	0.8218
3	100	0.8437
3	200	0.8579
4	50	0.8366
4	100	0.8495
4	200	0.8568
5	50	0.8531
5	100	0.8904
5	200	0.8907
6	50	0.8527
6	100	0.8738
6	200	0.8882

Table 8 presents a comparison between the final model results and the results of the support vector machine (SVM) and decision tree methods (traditional machine learning algorithms). As the table reveals, compared to the traditional machine learning algorithm, the CNN algorithm constructed in this study improved the relational extraction by approximately 10 percentage points. Therefore, this model was selected to build the relation extraction system. We also compared our results with the state-of-the-art language model BERT with a classifier. The results indicated that our CNN model performed almost the same as BERT; however, our model was much smaller (approximately 1000 parameters versus tens of thousands of parameters for BERT).

**Table 8:** Result of relation extraction and model comparison

Model	F1 Score
Our method (CNN)	0.8904
CNN w/o context bridge	0.8612
SVM	0.8196
Decision tree (4.5)	0.7956
BERT (SVM-classifier)	0.8918

### Knowledge fusion performance

**Fig. 14:**
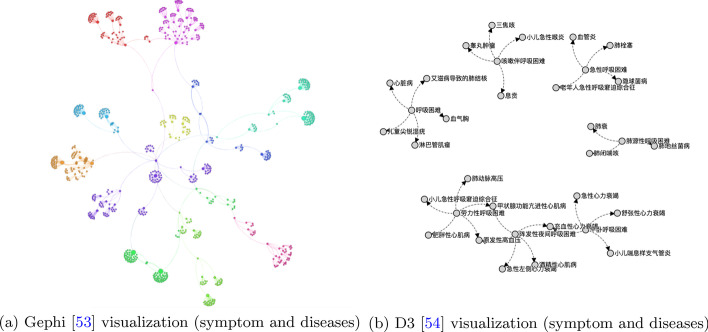
Extraction visualization

Knowledge fusion technologies improve the quality of a knowledge graph (statistics on automatic and manual work are provided in Table 9). As the final step of knowledge mining, we first merged similar entities (alignment) and solved conflicts between relations. The knowledge sizes before and after this process of entity alignment are provided in Table 10, while the precision (proportion of alignments manually evaluated as correct) is presented in Table 11, from which we can see that the co-training method performed better than the pre-training method. The reason for this result is that the co-training method trains word vectors together with the deep semantic extractor (CRF+Bi-LSTM); thus, the vectors express the semantic difference in the EHR. Specifically, the vectors in co-training imply the semantics of Chinese characters and the possible segmentation between them, which is not included in the pre-training method. The knowledge sizes before and after the consistency analysis process are presented in Table 12. The entities and relations decreased slightly. The quality of the knowledge graph was evaluated (due to the cost of evaluation, we did not evaluate the immediate results of entity alignment and conflict resolution), and the results are presented in Table 13, where accuracy refers to the correctness of the extracted tuples, while coverage represents the proportion of entities of a given medical encyclopedia that are covered by the extracted entities (here, we crawled the entities from 39 Health Network’s five semantic categories and used a crowdsourcing service to verify them, and the final test set included 36,285 entities). After knowledge fusion, we stored the knowledge graph in a database, and the visualization results are illustrated in Fig. 14, while some knowledge fusion results are presented in Additional file 1: Appendix 6.

**Table 9:** Automatic and manual knowledge fusion

Fused:	Entity (auto.)	(man.)	Relation (auto.)	(man.)
Count	5372	6357	918	665
Ratio	0.456	0.544	0.580	0.420

**Table 10:** Entity types and quantities before/after alignment

Entity:	Disease	Symptom	Treat	Test	Result
Before	11,963	17,812	4189	1052	10,065
After	7396	12,570	3764	1026	8641

**Table 11:** Entity alignment precision

Method	Precision (%)
Pre-trained	58.2
Co-trained	63.1

**Table 12:** Relation quantities before/after solving conflicts

Relation	Disease-symptom	Disease-treat	Disease-result	Test-result
Before	11,024	3209	5417	1028
After	9581	3197	5316	1001

**Table 13:** Results of quality assessment

Items	Results
Accuracy	0.8850
Coverage	0.9204

### Question answering performance

#### QA application demonstration

We developed a QA application that includes a search engine that uses Apache Solr [55] and our knowledge graph, and includes the QA model by combining it with the MemNN. With the KB, this application can provide relevant structured search results and native language answers promptly.

**Fig. 15:**
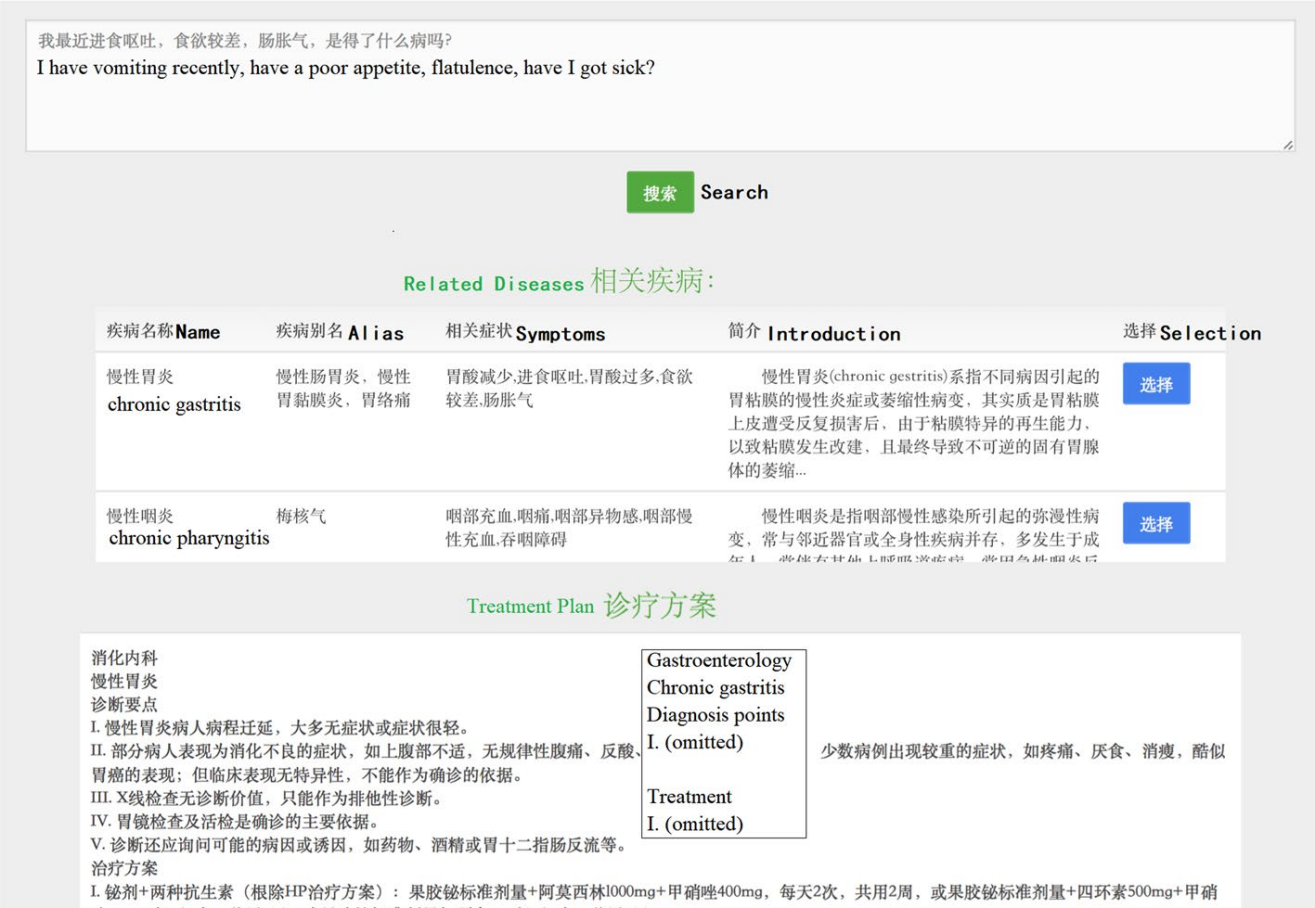
Example of semantic search results. Chronic nephritis

Figure 15 presents the semantic search results in the application system. The user entered various symptoms and wished to query for possible illnesses. The semantic search model mapped the user input search request to the entity “chronic gastritis” in the knowledge graph and provided a treatment plan for chronic gastritis.

**Fig. 16:**
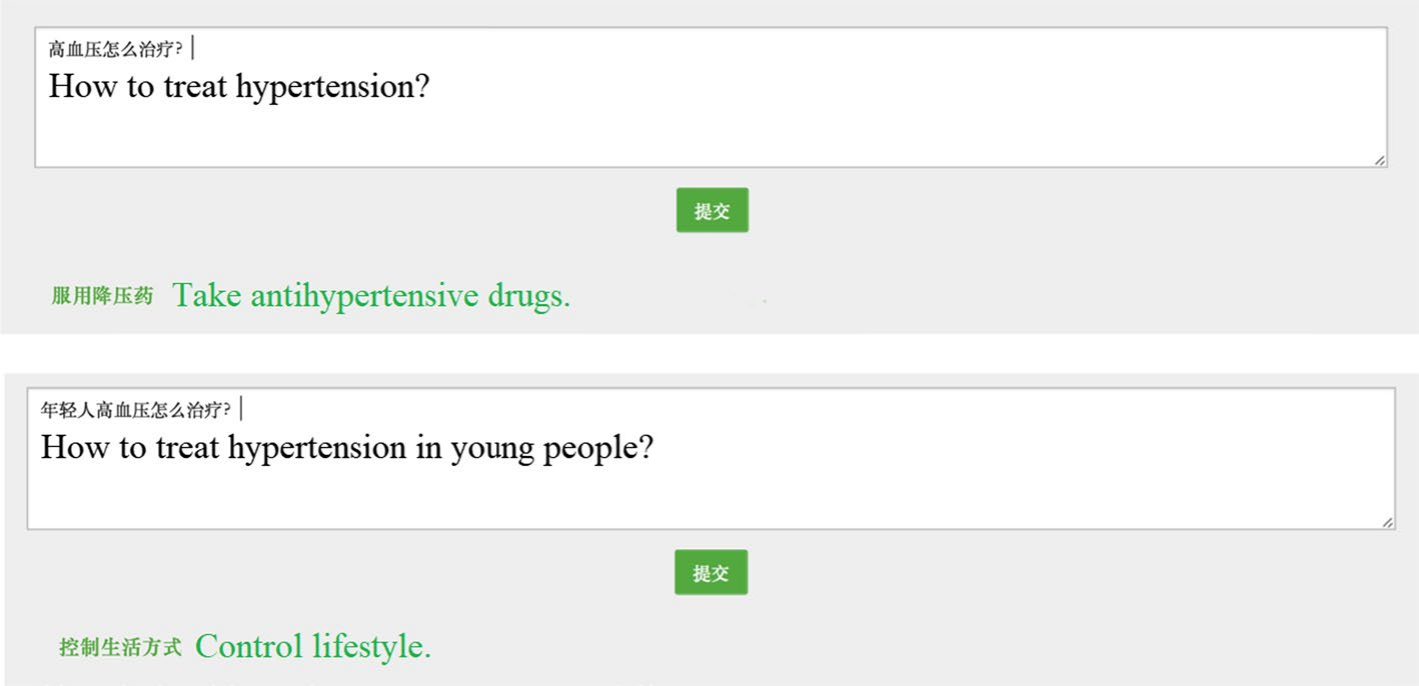
Example of a question answering (QA) task. A similar question with different contexts leads to different answers

Figure 16 presents several examples of an intelligent question and answer in the application system. The user entered two similar questions; however, due to the addition of a modifier in the second question, the QA system successfully recognized the semantic difference and provided different answers: taking medicine is a common answer, and modifying one’s lifestyle is directed at young persons (method in traditional Chinese medicine). Additional examples are provided in Additional file 1: Appendix 7.

#### QA quality evaluation

During the QA quality verification, we measured the correctness of single-fact questions (i.e., questions for a single disease or treatment). During the evaluation, we used a subset of the internet QA data as test data. As illustrated in Fig. 2, the title of a document in the QA corpus was a simple question of a patient, and the document content was the answer to the question.

##### Labeling test data

For evaluation, labeled question–answer pairs were required as an evaluation standard. If we extracted entities from titles and manually marked them to articles, considerable manpower would be necessary. Therefore, the target entity was annotated according to predefined rules mentioned in the Input Processing section.

Then, we extracted the entity in articles. The entity type that was consistent with the user’s intention and that appeared most frequently was recognized as the target entity (i.e., answer). In the actual QA data, from only part of the data, we were able to extract the intent category and target entity. For further convenience of testing, we only retained the part of the data that contained only one type of relation in its content by running relational extraction on the articles. For example, if the title was “what is the disease when I have fever and cough?” it was first recognized that the question pertained to a *disease*. Then, the NER was used on the title, which indicated that the question contained *symptoms*. Answers were then generated that represented the relation of the *symptoms* and *disease*. In the actual QA data, only a small part of the data met the requirements; thus, the final test data were considerably smaller than the original data which can be manually verified, as illustrated in Table 14.

**Table 14:** Processed question answering (QA) data for experiment

Entities	Disease	Treat	Symptom	Result	Test	Total
Amount	8753	9631	3186	1764	357	23,691

###### Experiment and results

From the processed data, we combined the question details and answers to constitute a question–answer pair as the training set and used it to train the MemNN-based QA model, as described in the last section.

Although it is difficult to compare the proposed method to other methods due to the cost of the implementation and data preparation, we implemented several methods from other studies for comparison. The LSTM was always used as a baseline [41], and Bi-LSTM always produced superior results. Thus, we compared our results to those of Bi-LSTM and an extension of Bi-LSTM that introduced an attention mechanism [56], which had better answer-selection performance.

We sent the questions from the test data to the system to measure the average accuracy. The algorithm performance was evaluated using the F1 score and precision on the top in the returned list (P@1) value; the results are presented in Table 15. With the KB, the accuracy improved by 10%, with the standard deviation unaffected compared to the attention-based Bi-LSTM. It may thus be the case that the memory mechanism is higher than a pure attention mechanism for medical QA tasks in Chinese.

**Table 15:** Comparison of question answering (QA) models

Model	F1	F1-STD	P@1	P@1-STD
Bi-LSTM	0.521	0.037	0.556	0.056
Attention+Bi-LSTM	0.546	0.023	0.572	0.025
Only MemNN	0.583	0.031	0.604	0.028
Our model	0.639	0.024	0.661	0.023

These results also demonstrate that our system was comparable in some respects to other state-of-the-art systems for the Chinese language [1] (which was not strictly due to the difference in data and evaluation methods), in which the best P@1 was 66%. [1] built an enormous data corpus and used an advanced neural network to obtain high accuracy. Our methods are different in that we used a KB to improve a simple neural network that could also achieve high performance; that is, our system takes the MemNN model as the algorithm model of an intelligent question and answers in the application system. We do not believe that Chinese word tokenization is necessary because vectorization is used in QA.

Our results were also comparable to the English QA system MEANS [12], which also uses an extracted KB; however, in QA, MEANS does not use a DNN. Instead, it achieves very high precision using concept relaxation and more logical inference. We also believe that despite the cost, building a logical inference together with a KB can further improve the accuracy.

## Conclusion

There is misleading information regarding medical care, hospitals, and treatments in Chinese networks and search engines, and many illegal or unethical information providers attempt to control information channels between reasonable solutions and individuals who need them. This study was driven by the belief that we can help ordinary people obtain the information they require in their native language and eliminate noise created by scammers.

This study used expert and online data in the knowledge building and answer generation tasks. The critical problems solved in this study were the extraction of semi-automatic knowledge and the training of memory and attention models for QA. For the first problem, we found that if we controlled semi-supervised learning with the seed quality, the successive supervised algorithm had a non-degenerative quality in semantic extraction and classification. For the second problem, our architecture significantly improved the answers. In performance evaluation, we determined that using bootstrapping and supervised learning resulted in a high-quality medical KB from expert data. Using high-quality KB and deep learning methods, one can quickly build a high-performance QA system for medical QA tasks in Chinese. Finally, to help a more significant number of people, we also created a support system for real users and intend to make it available to the network soon.

In the future, we plan to implement additional improvements to the system to 1) solve the questions of users with multiple diseases as their queries, (2) observe how the results are improved by adopting more complex neural networks and more extensive data (such as in [1]), and (3) compare our mechanism to others in open contests (such as the medical track of TREC).

## Supplementary Information

**Additional file 1.** Details about the data and methods including examples.

## Data Availability

Both the electronic health records dataset and internet question-answering corpus are available on https://www.synapse.org/amkmc (login with account “Litton” and password “A00e00c01B12” and download in column “Files”).

## Abbreviations

QA: Question-Answering
CNN: Convolutional Neural Network
LSTM: Long Short-Term Memory
Bi-LSTM: Bi-directional Long Short-Term Memory
MemNN: Memory Neural Network
TREC: Text REtrieval Conference
KB: Knowledge Base
DNN: Deep Neural Network
EHR: Electronic Health-medical Record
NLP: Natural Language Processing
SVM: Support Vector Machine
CRF: Conditional Random Field

## Footnotes

1 The largest healthcare information website in China with tens of thousands of doctors as information providers and tens of millions of users, which supports QA, information searching, and an encyclopedia created by experts: http://www.39.net

2 The support value (which is called *frequency* in [47]) is defined as the ratio of the seed entities to the extracted entities (extraction set) with pattern *p*. Seeds are confirmed as correct entities (supporting set); thus, the ratio between the two reflects the correctness of the pattern.

3 The confidence function calculates the cluster confidence [48] of the target pattern *p*: ($\frac{|correct\,extractions\,of\,p|}{|all\,extractions\,of\,p|}$).

4 An electronic encyclopedia similar to Wikipedia provided by Baidu, one of the leading IT company in China: https://baike.baidu.com/

5 We evaluated the results with precision, not recall. A large seed set involves more candidate patterns, which involves more noise. Our pattern validation method is not powerful enough to filter noise with very high precision. Thus, when the initial seed set is larger, the precision is lower.

6 Both Fig. 9b and 9c are statistically significant. In 9b, the p-value between size 20 and size 100 is 0.022, while in c, the p-value of the threshold comparison is 0.02, 5.3E–7, 3.2E–6 for 0.7 vs. 0.8, 0.7 vs. 0.9, and 0.8 vs. 0.9, respectively.
