# Supplementary material for: Answering medical questions in Chinese using automatically mined knowledge and deep neural networks: an end-to-end solution
Source: BMC Bioinformatics. 2022 Apr 15;23:136. doi: 10.1186/s12859-022-04658-2 (PMC9013171; doi:10.1186/s12859-022-04658-2)
Supplement: Supplementary file 1 — Additional file 1. Details about the data and methods including examples. [file 12859_2022_4658_MOESM1_ESM.docx]

**Appendices**

Here we have listed seven parts of experiment data to demonstrate our work. **The original data is in Chinese, so the translation may not be totally accurate.** So we are sorry for any possible misunderstanding and confusion in reading.

**Appendix 1. Seeds of Entities**

A1.1 Diseases

| 肺结核 | tuberculosis |
| --- | --- |
| 伤寒 | Typhoid fever |
| 肠炎 | enteritis |
| 败血症 | septicemia |
| 病毒性心炎 | Viral carditis |
| 狂犬病 | Rabies |
| 寄生虫感染 | Parasitic infection |
| 淋巴瘤 | Lymphoma |
| 帕金森 | Parkinson |
| 白血病 | leukemia |
| AIDS | AIDS |
| 恶性肿瘤 | Malignant tumor |
| 乳腺癌 | Breast cancer |
| 贫血 | anemia |
| 骨髓坏死 | Bone marrow necrosis |
| 肉芽肿 | Granulomatous |
| 甲状腺机能亢进症 | Hyperthyroidism |
| 胃溃疡 | Gastric ulcer |
| 结膜炎 | Conjunctivitis |
| 甲沟炎 | Paronychia |

A1.2 Treatment and Medicine

| 阿司匹林片 | Aspirin tablets |
| --- | --- |
| 奥美沙坦酯 | Olmesartan medoxomil |
| 阿莫西林胶囊 | Amoxicillin capsule |
| 氨非咖敏片 | Amphetamine |
| 比阿培南 | Biapenem |
| 百日咳片 | Whooping cough tablets |
| 补中益气丸 | Bu Zhong Yi Qi Wan |
| 布洛芬胶囊 | Ibuprofen capsules |
| 巴比妥口服 | Barbitur |
| 板蓝根冲剂 | Banlangen granules |
| 复方氨基酸注射液 | Compound Amino Acid Injection |
| 谷氨酰胺 | Glutamine |
| 硝酸甘油 | Nitroglycerin |
| 普拉洛芬 | Pralofen |
| 塞替派注射液 | Thiotepa injection |
| 白内障手术 | Cataract surgery |
| 肿瘤切除术 | Tumor resection |
| 土霉素片 | Oxytetracycline tablets |
| 抗逆转录疗法 | Antiretroviral therapy |
| 狂犬疫苗注射 | Rabies vaccine |

A1.3 Symptoms

| 喉痛 | Sore throat |
| --- | --- |
| 呼吸困难 | Difficulty breathing |
| 腰背刺痛 | Low back pain |
| 血沉增快 | Increased ESR |
| 清晨咳痰 | Early morning sputum |
| 周期性发热 | Periodic fever |
| 上腹痛 | Abdominal pain |
| 咯血 | Hemoptysis |
| 水肿 | Edema |
| 厌食 | Anorexia |
| 鼻咽部干燥感 | Nasopharyngeal dryness |
| 晕厥 | Syncope |
| 畏寒 | Chill |
| 前列腺疼痛 | Prostate pain |
| 排尿不尽感 | Endless urination |
| 肢体麻木 | Limb numbness |
| 恶心 | nausea |
| 尿频 | Frequent urination |
| 恐水 | Fear of water |
| 口唇苍白 | Pale lips |

A1.4 Test

| 脑电图 | EEG |
| --- | --- |
| 白细胞 | leukocyte |
| 中性粒细胞 | Neutrophil |
| 中性粒细胞比例 | Neutrophil ratio |
| 淋巴细胞比例 | Lymphocyte ratio |
| VAS评分 | VAS score |
| CT平扫 | CT plain scan |
| CT增强 | CT enhancement |
| 心超 | Heart B-ultrasound |
| 肺CTA | Pulmonary CTA |
| CK(pan) | CK (pan) |
| 肠鸣音 | borborygmus |
| 神经系统检查 | Neurological examination |
| X线 | X-ray |
| 体温 | body temperature |
| 心电图 | ECG |
| 血压 | blood pressure |
| 碱性磷酸酶 | Alkaline phosphatase |
| 总胆红素 | Total bilirubin |
| 核磁共振 | Nuclear magnetic resonance |

A1.5 Test results

[NUM] means that we normalized the test results that contain numbers as result and using string ‘[NUM]’ to replace any numbers for a unified expression.

| [NUM]% | [NUM]% |
| --- | --- |
| + | + |
| - | - |
| 阴性 | negative |
| 阳性 | positive |
| [NUM] | [NUM] |
| 肝功能不全 | Liver dysfunction |
| 血糖升高 | Elevated blood sugar |
| [NUM]*[NUM]/L | [NUM] * [NUM] / L |
| 双肺多发结节 | Multiple lung nodules |
| 浅表淋巴结肿大 | Superficial lymphadenopathy |
| 双肺呼吸音粗 | Thick lung sounds |
| [NUM]mmhg | [NUM] mmhg |
| 肿瘤细胞 | Tumor cell |
| 未见肿瘤细胞 | No tumor cells |
| 子宫缺如 | Absence of uterus |
| 左室壁增厚 | Left ventricular wall thickening |
| 血压升高 | High blood pressure |
| ++ | ++ |
| +++ | +++ |

**Appendix 2. Seeds of Relations**

A2.1 Symptom-Disease

| 听力下降, 分泌性中耳炎 | Hearing loss, secretory otitis media |
| --- | --- |
| 双眼视物模糊, 双眼白内障 | Blurred vision in both eyes, cataracts in both eyes |
| 呕血, 消化道出血 | Hematemesis, gastrointestinal bleeding |
| 泡沫尿, 肾功能异常 | Foamy urine, abnormal renal function |
| 心悸, 高血压 | Palpitations, hypertension |
| 小便淡红色, 肾结石 | Peeing pink, kidney stones |
| 腹痛, 结肠多发息肉 | Abdominal pain, polyp of the colon |
| 反酸, 慢性非萎缩性胃炎 | Acid reflux, chronic non-atrophic gastritis |
| 胸闷气促, 冠心病 | Chest tightness, shortness of breath, coronary heart disease |
| 咽痛, 肺炎 | Sore throat, pneumonia |
| 腰部疼痛, 腰椎间盘突出症 | Lumbar pain, lumbar disc herniation |
| 血尿, 肾病综合征 | Hematuria, nephrotic syndrome |
| 黑便, 肝硬化 | Melena, cirrhosis |
| 放射痛, 皮下神经痛 | Radiation pain, subcutaneous neuralgia |
| 嗳气, 十二指肠病变 | Radon, duodenal disease |
| 腰部疼痛, 骨质酥松 | Waist pain, flabby bone |
| 大便后出血, 直肠癌 | Bleeding after stool, rectal cancer |
| 心脏杂音, 房间隔缺损 | Cardiac murmur, atrial septal defect |
| 黄白色痰, 胸腔积液 | Yellowish white sputum, pleural effusion |
| 腰背疼痛, 腰椎骨折 | Back pain, lumbar fracture |

A2.2 disease-treatment/medicine

| 脑梗死 阿司匹林片 | Cerebral infarction, aspirin tablets |
| --- | --- |
| 分泌性中耳炎 手术治疗 | Secretory otitis media, surgical treatment |
| 双眼白内障 白内障手术 | Cataracts binocular, cataract surgery |
| 消化道出血 生长抑素止血 | Gastrointestinal bleeding, somatostatin |
| 肾功能异常 降压 | Renal dysfunction, hypotension |
| 肺腺癌 GP方案 | Lung adenocarcinoma, GP protocol |
| 高血压 氯沙坦钾片 | Hypertension, losartan potassium tablets |
| 痛经 中药治疗 | Dysmenorrhea, Chinese medicine treatment |
| 子宫肌瘤 手术治疗 | Uterine fibroids, surgery |
| 肺炎 泰能 | Pneumonia, imipenem cilastatin sodium for injection |
| 关节痛 保泰松 | Joint pain, phenylbutazone |
| 糖尿病 甘舒林 | Diabetes, Gansuline |
| 腰椎间盘突出症 镇痛 | Lumbar disc herniation, analgesia |
| 肾病综合征 激素治疗 | Nephrotic syndrome, hormone therapy |
| 十二指肠病变 制酸保胃 | Duodenal disease, Inhibit acidity to protect stomach |
| 冠心病 波立维 | Coronary heart disease, Polyvi |
| 前列腺增生 保列治 | Benign prostatic hyperplasia |
| 骨质酥松 钙片 | Bone Crispy, Calcium |
| 多发性骨髓瘤 PCD方案 | Multiple myeloma, PCD protocol |
| 原发性肝癌 肝脏切除术 | Primary liver cancer, liver resection |

A2.3 disease-test result

| 分泌性中耳炎, 鼓膜内陷 | Secretory otitis media, tympanic invagination |
| --- | --- |
| 膝关节退变, 胫骨上段骨质密度异常 | Knee joint degeneration, abnormal bone density in the upper tibia |
| 尿毒症, 肾功能异常 | Uremia, abnormal renal function |
| 肺腺癌, NapsinA（+） | Lung adenocarcinoma, NapsinA (+) |
| 肾结石, 左肾上段输尿管扩张 | Kidney stones, ureteral dilatation of the upper left kidney |
| 结肠多发息肉, 结肠上皮轻度异型增生 | Multiple polyps of the colon, mild dysplasia of the colonic epithelium |
| 慢性非萎缩性胃炎, 胃表面糜烂 | Chronic non-atrophic gastritis, gastric surface erosion |
| 子宫肌瘤, 子宫明显包块 | Uterine fibroids, marked mass in the uterus |
| 肺炎, 肺感染灶 | Pneumonia, focus of inflection on lung |
| 腰椎间盘突出症, 椎间盘混合性突出 | Lumbar disc herniation, mixed disc herniation |
| 股骨头坏死, 高密度灶 | Femoral head necrosis, high-density foci |
| 冠心病, 其余冠脉轻度病变 | Coronary heart disease, minor coronary lesions |
| 多发性骨髓瘤, 浆细胞骨髓瘤改变 | Multiple myeloma, plasma cell myeloma changes |
| 直肠癌, 直肠不规则肿物 | Rectal cancer, Irregular mass on rectal |
| 原发性肝癌, 肝多枚结节灶 | Primary liver cancer, multiple nodules of the liver |
| 前列腺癌, PSA增高 | Prostate cancer, increased PSA |
| 黄疸, 肝内胆管扩张 | Jaundice, intrahepatic bile duct dilatation |
| 淋巴瘤, 淋巴组织增生 | Lymphoma, lymphoid hyperplasia |
| 白内障, 玻璃体混浊 | Cataract, opacification |
| 口腔粘膜损害, 口腔白斑块 | Oral mucosal damage, oral white plaque |

A2.4 test-test_result

| 体温, [NUM]℃ | Body temperature, [NUM]℃ |
| --- | --- |
| 血红蛋白, [NUM]g/L | Hemoglobin, [NUM]g/L |
| 尿蛋白, +++ | Urine protein, +++ |
| 胃镜, 胃体多发息肉 | Gastroscope, polyp of body |
| 脉搏, [NUM]次/分 | Pulse, [NUM]times/minute |
| 血压, [NUM]mmHg | Blood pressure, [NUM]mmHg |
| 妇科B超, 局部压迫内膜 | Gynecological B-ultrasound, local compression of endometrium |
| 冠脉CTA, 冠脉血管严重狭窄 | Coronary CTA, severe coronary stenosis |
| 胸部CT, 右侧肺气囊 | Chest CT, right lung balloon |
| 血白细胞, [NUM]*[NUM]/L | White blood cells, [NUM]*[NUM]/L |
| 血糖, [NUM]mmol/L | Blood glucose, [NUM]mmol/L |
| VAS评分, [NUM]分 | VAS score, [NUM]points |
| 神经系统检查, - | Neurological examination,- |
| 甲状腺触诊, 明显肿大及结节 | Palpation of thyroid, marked enlargement and nodules |
| 腹部CT, 肝硬化 | Abdominal CT, cirrhosis |
| 隐血, +++ | Occult Blood, +++ |
| 动静脉CTA, 门脉主干附壁血栓形成 | Arteriovenous CTA, portal vein mural thrombosis |
| 单核细胞（%）, [NUM]% | Monocytes (%), [NUM]% |
| HBV-DNA, [NUM]/ml | HBV-DNA, [NUM]/ml |
| 超敏C反应蛋白, [NUM]mg/L | High-sensitivity C-reactive protein, [NUM]mg/L |

**Appendix 3. Demonstration of Bootstrapping & Extraction**

A3.1 Demonstration for Entity Extraction

The Entity Extraction Workflow includes EIGHT steps below:

**STEP 1:** Build seed words: 20 seeds for a semantic type, for example a seed for disease is “upper respiratory infection” (上呼吸道感染)；

**STEP 2:** Match the seeds in the whole corpus, and gets the characters or symbols in left and right sides of the words as candidate patterns.

For example, the following text contains the seed word, which is bolded, and the clause is underlined.

患儿2天前在家中无明显诱因下出现发热，为不规则热，体温波动于39.1℃~37.7℃，无寒战，无抽搐，无嗜睡，无昏迷；有少许流涕，无咳嗽，无气促，无阵发性哭闹不安，有恶心呕吐1次，为哭吵后呕出较多奶汁，无腹泻，无粘液血便。起病后家属自行予以“泰诺林混悬液”口服多次退热处理，予“芙朴感冒颗粒”口服治疗1次，患儿今仍有发热，测体温38.8℃，无咳嗽，无气促。故来本院门诊就诊，予以“小儿布洛芬栓1/2粒”塞肛处理后，为进一步诊治拟“**上呼吸道感染**”收住入院。自发病来，患儿神志清，精神可，纳乳如常，大小便正常，体重无变化。

In English:

The child had fever without obvious inducement at home 2 days ago. It was irregularly hot, body temperature fluctuated between 39.1 ℃ ~ 37.7 ℃, no chills, no convulsions, no lethargy, no coma; a little runny, no cough, no gas No paroxysmal crying, no nausea and vomiting, vomiting more milk after crying, no diarrhea, no mucus and bloody stools. After the onset of the illness, the family members were given oral "Tylinin Suspension" for several times of heat treatment, and "Fupu Ganmao Granules" were given oral treatment once. The child still had fever, and his body temperature was 38.8 ℃. He had no cough and shortness of breath. Therefore, after visiting the outpatient clinic of our hospital, the child was treated with "1/2 capsules of ibuprofen suppository", and was admitted to the hospital for further diagnosis and treatment of "**upper respiratory infection**". Since the onset, the child has a clear mind, a good spirit, normal breast feeding, normal bowel movements, and no change in weight.

In the clause“为进一步诊治拟‘上呼吸道感染’收住入院。”, we can extract 15 candidate patterns, as followings:

A）诊治拟“<dis>”收住入

B）治拟“<dis>”收住入

C）拟“<dis>”收住入

D）“<dis>”收住入

E）诊治拟“<dis>”收住

F）治拟“<dis>”收住

G）拟“<dis>”收住

H）“<dis>”收住

I）诊治拟“<dis>”收

J）治拟“<dis>”收

K）拟“<dis>”收

L）“<dis>”收

M）诊治拟“<dis>”

N）治拟“<dis>”

O）拟“<dis>”

**STEP 3.** Matching text in the corpus with the candidate patterns. For example, with pattern “拟‘<dis>’收住”, we can match a string like “支原体感染” (mycoplasma infection) from a text “门诊拟’支原体感染’收住入院。” (admitted to hospital with ‘mycoplasma infection’).

**STEP 4.** Calculate support and confidence scores for each candidate patterns. If a candidate pattern, e.g. 治拟”<dis>”收住 extracts 9 words: “支原体感染”(new word), ”颅内感染”(disease seed), ”右肺占位”(test-result seed), “脑梗死”(disease seed), “肝功能异常”(test-result seed), “骨折”(disease seed), “多发性骨髓瘤”(disease seed), “高血压病”(disease seed), “糖尿病肾病”(disease seed)) in STEP 3, in which 8 are seed words (not necessarily be the initial seeds; “支原体感染” is a new word and others are already in seed set) and 6 are entity words from encyclopedia with target semantic type (disease), then the supporting score of this pattern is 8/9, and confidence is 6/8. The weighted sum is 0.785 > threshold 0.7, so the candidate pattern is validated.

**STEP 5.** The words extracted with validated patterns are called “candidate seeds”. For example the pattern 治拟“<dis>”收住can match new words “肺炎” (pneumonia) and ”高血压” (hypertension), which are “candidate seeds”.

**STEP 6.** Validate a candidate seed by searching it in an electronic encyclopedia. If the candidate seed matches an entry of the encyclopedia (like the following figure shows), it will be a “new seed”. If not matched, it will be manually checked.


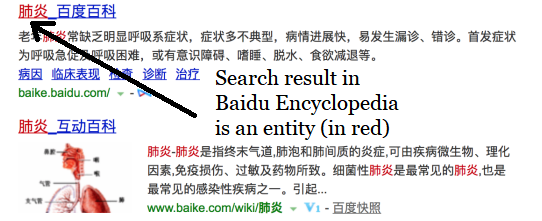


**STEP 7**. Repeat Step 1-6 until no new seed is generated. Then we tag text: tagging characters in recognized entities with semantic tag like ‘S-DIS’, ‘M-DIS’ and ‘E-DIS’ (stating, middle, and ending character for disease), and tagging characters not in entities with ‘O’ tag.

**STEP 8.** Use the tagged text as training data to train BI-LSTM+CRF model, and get the NER model.

**Result**: from the text above, the extracted entities includes diseases, symptoms, treatment (or medicine), test, result. **And the word/phrase with a (*) superscript is matched from encyclopedias and a (^) is for manual (or rule-based) checking. The brackets show the nested relations inside an entity.**

The child had fever without obvious inducement at home 2 days ago. It was irregularly hot, (body temperature)* fluctuated (between (39.1℃)^-(37.7℃)^)^, (no chills*)^, (no convulsions*)^, (no lethargy*)^, (no coma*)^; a little runny*, (no cough*)^, (no (short breath)*)^, (no paroxysmal*)^ crying^, (no nausea*)^ and vomiting, vomiting milk after crying, (no diarrhea*)^, (no (mucus* and (bloody stools)*)^)^. After the onset of the illness, the family members were given oral “(tylinin Suspension)*" for several times of heat treatment, and "(Fupu Ganmao Granules)*" were given oral treatment once. The child still had fever, and his body temperature was (38.8 ℃)^. He had (no cough*)^ and short breath. Therefore, after visiting the outpatient clinic of our hospital, the anus was treated with "(1/2 capsules of (ibuprofen suppository)* )^", and was admitted to the hospital for further diagnosis and treatment of "upper respiratory infection (seed)". Since the onset, the child has a clear mind, a good spirit, normal breast feeding, normal bowel movements, and no change in weight.

A3.2 The numbers of encyclopedia matching

| Entity: | disease | symptom | treat/drug | test | result |
| --- | --- | --- | --- | --- | --- |
| Total number | 11963 | 17812 | 4189 | 1052 | 10065 |
| Matched | 10965 | 10742 | 3881 | 966 | 5134 |

A3.3 Demonstration for Relation Extraction

Same to Entity Extraction, the Relation Extraction also has eight steps:

**STEP 1:** Build seed relations: 20 seeds for a semantic type, for example a seed for disease-treatment is <“上呼吸道感染upper respiratory infection”, dis-tre, “小儿布洛芬ibuprofen suppository”>.

**STEP 2:** Match the seeds in the whole corpus, and get **the substrings between the words**. Given the same example as A7.1, in the text “予以‘小儿布洛芬栓1/2’塞肛处理后，为进一步诊治拟‘上呼吸道感染’收住入院。” (the child was treated with "1/2 capsules of ibuprofen suppository", and was admitted to the hospital for further diagnosis and treatment of "upper respiratory infection), after filtering string words with a stop-list, 7 candidate patterns (substrings) are extracted by composing substrings of the string between the two entities in the seed relation.

A）<treat>塞肛<dis>

B）<treat>处理<dis>

C）<treat>诊治<dis>

D）<treat>塞肛 处理<dis>

E）<treat>处理 诊治<dis>

F）<treat>塞肛 诊治<dis>

G）<treat>塞肛 处理 诊治<dis>

**STEP 3.** Matching text in the corpus with the candidate patterns, and get candidate relations.

**STEP 4.** Validate the patterns with the extracted candidate relations. Given a pattern “<treat>处理<dis>” gets 32 candidate relations, 8 of which contains a pair of valid entities, and 6 of which contains corrected semantic type of the entities, supporting is 1/4, confidence 3/4, weighted sum is the validation score = 0.625 < 0.7, so discard this pattern.

**STEP 5.** The word pairs extracted with validated patterns are called “candidate seeds”.

**STEP 6.** Validate a candidate seed by searching it in search engine. If the entities of a candidate seed appears in at least 8 of the top 10 search results, it will be validated as a “new seed”. If not matched, it will be manually checked.

**STEP 7**. Repeat Step 1-6 until no new seed is generated. Then we tag text: tagging characters between the entity pair in a relation, with semantic tag like ‘S-DIS-TRE’, ‘M-DIS-TRE’ and ‘E-DIS-TRE’ (stating, middle, and ending character for disease-treatment relation), and tagging characters not in relation with ‘O’ tag.

**STEP 8.** Use the tagged text as training data to train CNN model, and get the relation extraction model.

**Appendix 4. Entities Evaluated**

Here we list the randomly selected 200 extracted entities for the model parameter selection for entity extraction. In order to reduce burdens of checking, we first randomly select 100 documents for relation extraction, and randomly labeled 200 entities (ground truth) from them. Bolded words are incorrect in all models. For demonstrative convenience, only correctness is considered. The precision and recall calculation is not included since it is too complicated (different for each model).

There are five combinations of parameters in the experiment (CRF-pretrain, BI-LSTM-pretrain, CRF-BI-LSTM-pretrain, , BI-LSTM-cotrain, CRF-BI-LSTM-cotrain), so we list the performance like ‘x,x,x,x,x’, x can be ‘y’ (correct), ‘n’ (incorrect) and ‘m’ (missing).

| **Extracted** | **English** | **model_correctness** | **Type** |
| --- | --- | --- | --- |
| 鼻中隔溃疡 | Nasal septum ulcer | y,y,y,y,y | disease |
| 胫骨结核 | Tibial tuberculosis | y,y,y,y,y | disease |
| 裂谷热 | Rift Valley Fever | n,n,y,n,y | disease |
| 咽部肿物 | Pharynx | y,y,y,y,y | disease |
| 肾上腺坏死 | Adrenal necrosis | m,m,y,m,y | disease |
| 贾第虫病 | Giardiasis | n,n,y,y,y | disease |
| 咽结膜热 | Conjunctival fever | y,y,y,y,y | disease |
| 继发性贫血 | Secondary anemia | y,y,y,y,m | disease |
| 视物变形 | Vision distortion | y,y,y,y,y | disease |
| 德戈病 | Dego disease | y,y,y,y,y | disease |
| 巴尔通体病 | Barton body disease | y,y,y,y,y | disease |
| 蜡油样骨病 | Waxy osteopathy | y,y,y,y,y | disease |
| 鼻腔肿物 | Nasal mass | n,y,y,y,y | disease |
| 阴道腺病 | Vaginal adenopathy | y,m,y,y,y | disease |
| 黑布拉痒疹 | Pruritus heibra | y,y,y,y,y | disease |
| 狼疮性脑病 | Lupus encephalopathy | m,y,y,y,y | disease |
| 支气管息肉 | Bronchial polyps | y,y,y,y,y | disease |
| 涎腺炎 | Salivary glanditis | m,m,m,y,y | disease |
| 癫痫 | epilepsy | y,y,y,y,y | disease |
| 阴茎炎 | Penile inflammation | y,y,y,y,y | disease |
| 尿道脱垂 | Urethral prolapse | m,m,y,y,y | disease |
| 视交叉疾患 | Optic cross disease | y,y,y,y,y | disease |
| 面神经麻痹 | Facial nerve palsy | y,y,y,y,y | disease |
| 肩蜂窝织炎 | Shoulder Cellulitis | y,y,y,y,y | disease |
| **肾囊肿** | **Renal cyst** | n,n,n,n,n | disease |
| 视乳头前膜 | Anterior papillary membrane | y,y,y,y,y | disease |
| 先天性巨胃 | Congenital giant stomach | y,y,y,y,y | disease |
| 脊柱后侧凸 | Scoliosis | y,y,y,y,y | disease |
| 网膜囊肿 | Omental cyst | y,y,y,y,y | disease |
| 子宫壁妊娠 | Uterine wall pregnancy | y,y,y,m,y | disease |
| 前房角粘连 | Anterior chamber adhesions | n,n,y,n,y | disease |
| 声带麻痹 | Vocal cord paralysis | y,y,y,y,y | disease |
| 额骨骨疣 | Frontal bone warts | y,y,y,y,y | disease |
| 甲状舌管瘘 | Thyroglossal fistula | y,m,y,y,y | disease |
| 急性牙髓炎 | Acute pulpitis | y,y,y,y,y | disease |
| 结肠原位癌 | Colon carcinoma in situ | m,y,y,n,y | disease |
| 颈段脊膜瘤 | Cervical meningiomas | y,y,y,y,y | disease |
| 阴唇粘连 | Labial adhesions | y,y,y,y,y | disease |
| **二尖瓣狭窄** | **mitral stenosis** | n,n,n,n,n | disease |
| 外阴疤痕 | Vulvar scar | y,y,y,y,y | disease |
| 骨质疏松 | Osteoporosis | y,y,y,y,y | disease |
| 假两性畸形 | Pseudohermaphroditism | y,y,y,y,y | disease |
| 滑膜增生 | Synovial hyperplasia | y,y,y,y,y | disease |
| 视网膜结核 | Retinal tuberculosis | y,y,y,y,m | disease |
| 附睾阴囊瘘 | Epididymal scrotal fistula | y,y,y,y,y | disease |
| 脑白质病 | White matter disease | y,n,y,y,y | disease |
| 生殖器萎缩 | Genital atrophy | y,y,y,y,y | symptom |
| 免疫力下降 | decrease in immunity | m,y,y,y,y | symptom |
| 发音不清 | Inarticulate | y,y,n,y,y | symptom |
| 怕冷 | Afraid of cold | y,y,y,y,y | symptom |
| 情绪不稳定 | unstable emotion | y,y,y,y,y | symptom |
| 粪便量少 | Feces are low | y,y,y,y,y | symptom |
| 强迫俯卧位 | Forced prone position | y,y,y,y,y | symptom |
| 失语症 | aphasia | y,y,y,y,y | symptom |
| 异常鱼鳞癣 | Abnormal ichthyosis | n,n,n,y,y | symptom |
| 低血糖症状 | Hypoglycemia symptoms | y,y,y,y,y | symptom |
| 精神失常 | Mental disorder | y,y,y,y,y | symptom |
| 尿中有泡沫 | Foam in the urine | y,y,y,y,y | symptom |
| 心境不良 | Bad mood | n,y,y,y,y | symptom |
| 皮肤疱疹 | Skin herpes | y,y,y,y,y | symptom |
| 舌乳头增生 | Hyperplasia of tongue | y,y,y,y,y | symptom |
| 腰背痛 | waist pain | y,y,y,y,y | symptom |
| 瘙痒感 | Itching | n,y,y,y,y | symptom |
| 流鼻血 | Nosebleed | m,m,m,y,y | symptom |
| 行为异常 | Abnormal behavior | y,y,y,y,y | symptom |
| 嗅觉倒错 | Smelling wrong | y,y,y,y,y | symptom |
| 极度口渴 | Extreme thirst | y,y,y,y,y | symptom |
| 厌奶 | Tired of milk | y,y,y,y,y | symptom |
| 面部潮红 | Facial flushing | y,y,y,y,y | symptom |
| 耳部胀满感 | Ear fullness | y,y,y,y,y | symptom |
| 脸裂 | Cracked face | n,n,y,y,y | symptom |
| 肚脐痒 | Navel itching | y,y,y,y,y | symptom |
| 小腿水肿 | Calf edema | y,y,y,y,y | symptom |
| 关节强硬 | Strong joints | y,y,y,y,y | symptom |
| 股后疼痛 | Post-femoral pain | y,y,y,y,y | symptom |
| 腹部沉重 | Heavy belly | y,y,y,y,y | symptom |
| 中骨盆狭窄 | Pelvic stenosis | y,y,y,y,y | symptom |
| **球蛋白异常** | **Globulin abnormalities** | n,n,n,n,n | symptom |
| **脉数** | **Pulse number** | n,n,n,n,n | symptom |
| 皮肤肿物 | Skin swelling | y,y,y,y,y | symptom |
| 药物性肥胖 | Drug-induced obesity | y,y,y,y,y | symptom |
| 斜形骨折 | Oblique fracture | y,y,y,y,y | symptom |
| 行走不便 | Inconvenience of walking | y,y,y,y,y | symptom |
| 内分泌紊乱 | hormone imbalance | y,y,y,y,y | symptom |
| 鼻孔闭锁 | Nostril atresia | y,y,y,y,y | symptom |
| 附睾硬结 | Epididymal sclerosis | y,y,y,y,y | symptom |
| 声音异常 | Sound abnormal | y,y,y,m,m | symptom |
| 替勃龙 | Tibolone | n,y,y,y,y | treat/drug |
| 肝囊肿切除术 | Liver cystectomy | y,y,y,y,y | treat/drug |
| 癣宁搽剂 | Ringworm tincture | y,y,y,y,y | treat/drug |
| 胜红清热片 | Shenghong Qingre (T) | y,y,y,y,y | treat/drug |
| 化疗 | Chemotherapy | m,m,y,y,y | treat/drug |
| 麝香解痛膏 | Musk pain relief cream (T) | y,y,y,y,y | treat/drug |
| 止血 | Hemostasis | y,y,y,y,y | treat/drug |
| 甲氨蝶呤 | Methotrexate | y,y,y,y,y | treat/drug |
| 手术治疗 | Surgical treatment | y,y,y,y,y | treat/drug |
| 氧化锌软膏 | Zinc oxide ointment | y,y,y,y,y | treat/drug |
| 苯甲酸钠 | sodium benzoate | y,y,y,y,y | treat/drug |
| 细辛脑 | Asarum | n,m,n,m,y | treat/drug |
| TIPS栓塞术 | TIPS embolization | y,y,y,y,y | treat/drug |
| 乳酸钙片 | Calcium lactate tablets | y,y,y,n,y | treat/drug |
| 肾移植术 | Kidney transplant | y,y,y,y,y | treat/drug |
| 感冒灵颗粒 | Cold spirit particles | y,y,y,y,y | treat/drug |
| 抗感染治疗 | Anti-infective treatment | y,y,y,y,y | treat/drug |
| 横结肠造瘘 | Transcolostomy | y,y,y,y,y | treat/drug |
| PG方案化疗 | PG regimen chemotherapy | y,n,m,n,y | treat/drug |
| 妥布霉素 | Tobramycin | y,y,y,y,y | treat/drug |
| 抗排异治疗 | Anti-rejection therapy | y,y,y,y,y | treat/drug |
| 结晶磺胺 | Crystalline sulfa | y,y,y,y,y | treat/drug |
| 雅施达 | Yashida(Perindopril) | y,y,y,y,y | treat/drug |
| 泊那替尼 | Ponatinib | y,y,y,y,y | treat/drug |
| 氨丁三醇 | Tromethamine | y,y,y,y,y | treat/drug |
| 氢氯噻嗪 | Hydrochlorothiazide | y,y,y,y,y | treat/drug |
| 预防感染 | Prevent infection | y,y,y,y,y | treat/drug |
| 培美曲塞 | Pemetrexed | y,y,y,y,y | treat/drug |
| CVAD方案化疗 | CVAD regimen chemotherapy | m,m,m,m,m | treat/drug |
| 肺癌根治术 | Radical lung cancer surgery | y,y,y,y,y | treat/drug |
| 头孢匹胺钠 | Cefpiramide Sodium | y,y,y,y,y | treat/drug |
| 冠心康胶囊 | Guanxinkang Capsules | y,y,y,y,y | treat/drug |
| 同位素治疗 | Isotope therapy | y,y,y,y,y | treat/drug |
| **对症治疗** | **Symptomatic treatment** | n,n,n,n,n | treat/drug |
| 人工晶体植入 | Intraocular lens implantation | y,m,m,y,y | treat/drug |
| 白内障手术 | Cataract surgery | y,y,y,y,y | treat/drug |
| 氢氯噻嗪 | Hydrochlorothiazide | y,y,y,y,y | treat/drug |
| 甲硝唑洗液 | Metronidazole lotion | y,y,y,y,y | treat/drug |
| 心脏彩超 | Echocardiography | y,m,y,y,y | test |
| 总胆红素 | Total bilirubin | y,y,y,y,y | test |
| 甲状腺超声检查 | Thyroid ultrasound | n,y,y,y,y | test |
| 直接胆红素 | Direct bilirubin | y,y,y,y,y | test |
| 总蛋白 | Total protein | m,y,m,y,y | test |
| 角膜内皮计数 | Corneal endothelium count | y,y,y,y,y | test |
| 荧光染色体原位杂交检查（FISH） | Fluorescent Chromosome In Situ Hybridization (FISH) | y,y,y,y,y | test |
| 碱性磷酸酶 | Alkaline phosphatase | y,y,y,y,y | test |
| 血糖检查 | blood sugar check | y,y,y,y,y | test |
| 上中下腹部CT平扫 | CT scan of upper, middle and lower abdomen | y,y,y,y,y | test |
| 癌胚抗原 | Carcinoembryonic antigen | n,y,n,y,y | test |
| 肌酐 | Creatinine | y,y,y,y,y | test |
| 神经系统检查 | Neurological examination | y,y,y,y,y | test |
| 尿酸 | Uric acid | y,y,y,y,y | test |
| 低密度脂蛋白-C4 | LDL-C4 | m,y,y,y,y | test |
| 总钙 | Total calcium | y,y,y,y,y | test |
| 总胆固醇 | Total cholesterol | y,y,y,y,y | test |
| 肺部增强CT | Lung enhanced CT | y,y,y,y,y | test |
| ALK-lung | ALK-lung | n,y,y,y,y | test |
| 纤支镜检查 | Bronchoscopy | y,y,y,y,y | test |
| 全腹CT | CT | y,y,y,y,y | test |
| **血压检查** | **Blood pressure** | n,n,n,n,n | test |
| 肺部CT | Lung CT | y,y,y,y,y | test |
| 白球蛋白比例 | Leucin ratio | y,y,y,y,y | test |
| 腺苷酸脱氨酶 | Adenylate deaminase | y,n,y,y,y | test |
| 谷氨酰转酞酶 | Glutamyltransferase | y,y,m,y,m | test |
| 支气管镜治疗 | Bronchoscopy | y,y,y,y,y | test |
| 穿刺病理 | Puncture pathology | y,y,y,y,y | test |
| 肝功能 | liver function test | y,y,y,y,y | test |
| 总胆汁酸 | Total bile acid | y,y,y,y,y | test |
| 胸部CT平扫 | CT scan of the chest | n,n,n,y,y | test |
| 磁共振扫描 | Magnetic resonance scan | y,y,y,y,y | test |
| 肝胆脾胰（彩超） | Liver, Gallbladder, Spleen and Pancreas (Color Doppler) | n,n,m,m,y | test |
| 胸部CT | Chest CT | y,y,y,y,y | test |
| 白蛋白 | albumin test | y,y,y,y,y | test |
| 白细胞计数 | White blood cell count | y,y,y,y,y | test |
| 妇检 | Gynecological examination | y,y,y,y,y | test |
| 高密度脂蛋白-CC | HDL-CC | y,y,m,m,m | test |
| 甘油三酯 | Triglyceride | y,y,y,y,y | test |
| 间接胆红素 | Indirect bilirubin | y,y,y,y,y | test |
| 全身骨显像 | Whole body bone imaging | y,y,y,y,y | test |
| 胸主动脉CTA | Thoracic aorta CTA | m,y,y,y,y | test |
| 谷丙转氨酶 | Alanine aminotransferase | y,y,y,y,y | test |
| MRI | MRI | y,y,y,y,y | test |
| NapsinA | NapsinA | m,y,y,y,y | test |
| 血小板计数 | Platelet count | y,y,y,y,y | test |
| 甲状旁腺激素 | Parathyroid hormone | y,y,y,y,y | test |
| 谷草转氨酶 | Aspartate aminotransferase | y,y,y,y,y | test |
| （+） | (+) | y,y,y,y,y | result |
| 食管上段异物嵌顿 | Incarcerated foreign body in upper esophagus | y,y,y,y,y | result |
| 肝内多发转移瘤考虑 | Multiple liver metastases considered | n,y,y,y,y | result |
| 食管中段异物 | Foreign body in middle esophagus | y,y,y,y,y | result |
| 左侧甲状腺可见低回声结节 | Hypoechoic nodules visible on left thyroid | m,y,y,y,y | result |
| PML/RARA基因t（15；17）[NUM]% | PML / RARA gene t (15; 17) [NUM]% | m,m,n,n,y | result |
| 右上叶支气管开口唇形狭窄 | Right upper lobe bronchial opening lip narrow | y,y,y,y,y | result |
| **ST-T未见明显异常改变** | **No obvious abnormal changes on ST-T** | n,n,n,n,n | result |
| **右眼 [NUM]/mm2，左眼 [NUM]/mm2** | **Right eye [NUM] / mm2, left eye [NUM] / mm2** | n,n,n,n,n | result |
| 阴道残端愈合欠佳 | Poor stump healing | y,y,y,y,y | result |
| [NUM]pg/ml | [NUM] pg / ml | y,y,y,y,y | result |
| 左心室舒张顺应性减低 | Reduced left ventricular diastolic compliance | n,n,n,m,y | result |
| 左乳结节 | Left breast nodule | y,y,y,y,y | result |
| [NUM]mmol/L | [NUM] mmol / L | y,y,y,y,y | result |
| 骨质代谢异常 | Abnormal bone metabolism | y,y,y,y,y | result |
| 肌酐升高 | Elevated creatinine | y,y,y,y,y | result |
| 右上肺肿块 | Upper right lung mass | y,y,y,y,y | result |
| 右侧甲状腺可见囊性结节 | Cystic nodule visible in right thyroid | n,y,y,y,y | result |
| **（-）** | **(-)** | m,m,m,m,m | result |
| [NUM]g/L | [NUM] g / L | n,m,n,y,y | result |
| 双侧甲状腺形态大小正常 | Normal bilateral thyroid morphology | n,n,y,y,y | result |
| [NUM]U/L | [NUM] U / L | y,y,y,y,y | result |
| 右膝半月板损伤 | Right knee meniscus injury | y,n,y,y,y | result |
| [NUM]umol/L | [NUM] umol / L | y,y,y,y,y | result |
| 左侧腹股沟疝 | Left inguinal hernia | y,y,y,y,y | result |
| **两侧侧脑室旁缺血灶** | **Paraventricular ischemic foci** | n,n,n,n,n | result |
| 右肺中叶近水平裂 | Right horizontal midlobed | n,y,y,y,y | result |

**Appendix 5. Relations Evaluated**

Here we list the randomly selected 200 extracted relations for the model parameter selection for relation extraction. In order to reduce burdens of checking, we first randomly select 30 documents for relation extraction, and randomly labeled 254 relations from them. Bolded relations are incorrect in all models. For demonstrative convenience, only correctness is considered. The precision and recall calculation is not included since it is too complicated (different for each model).

There are 12 combinations of parameters (4 CNN window sizes×3 word vector dimensions) in the experiment. For demonstrative convenience, we only compare CNN window sizes (3-6) under word vector dimensions of 200, so we list the performance like ‘x,x,x,x’, x can be ‘y’ (correct), ‘n’ (incorrect) and ‘m’ (missing). The relationship has several short names: dis-symp (disease and symptom), dis-tre (disease and treatment/drug), dis-test (disease and test), dis-ter (disease and test_result).

| Relation | English | model-correctness | Type |
| --- | --- | --- | --- |
| 脑梗,意识障碍 | Cerebral infarction, disturbance of consciousness | n,n,y,y | dis-symp |
| 脑梗,麻痹 | Cerebral infarction, paralysis | y,y,y,y | dis-symp |
| 脑梗,四肢瘫 | Cerebral infarction, quadriplegia | n,y,n,y | dis-symp |
| 脑梗,昏迷 | Cerebral infarction, coma | y,y,y,y | dis-symp |
| 脑梗,CT | Cerebral infarction, CT | y,y,y,y | dis-test |
| 脑梗,神经系统检查 | Cerebral infarction, nervous system examination | y,y,y,n | dis-test |
| 脑梗,MRI | Cerebral infarction, MRI | y,y,y,y | dis-test |
| 脑梗,超声检查 | Cerebral infarction, ultrasound | y,y,y,y | dis-test |
| 脑梗,血管狭窄 | Cerebral infarction, vascular stenosis | y,y,y,y | dis-result |
| 脑梗,动脉硬化 | Cerebral infarction, arteriosclerosis | y,y,y,y | dis-result |
| 超声检查,血管狭窄 | Ultrasound examination, vessel stenosis | n,y,y,y | test-result |
| 超声检查,动脉硬化 | Ultrasound, arteriosclerosis | n,y,y,n | test-result |
| 脑梗,降压 | Cerebral infarction, blood pressure | y,y,y,y | dis-tre |
| 脑梗,依达拉奉 | Cerebral infarction, edaravone | y,y,y,y | dis-tre |
| 脑梗,胞二磷胆碱 | Cerebral infarction, citicoline | n,n,y,y | dis-tre |
| 脑梗,手术治疗 | Cerebral infarction, surgical treatment | y,y,y,y | dis-tre |
| 脑梗,内支架 | Cerebral infarction, stent | y,y,y,y | dis-tre |
| 狂犬病,厌水 | Rabies | y,y,y,y | dis-symp |
| 狂犬病,恐惧 | Rabies, fear | y,y,y,y | dis-symp |
| 狂犬病,焦虑 | Rabies, anxiety | y,y,y,y | dis-symp |
| 狂犬病,易怒 | Rabies, irritable | y,y,y,y | dis-symp |
| 狂犬病,痉挛 | Rabies, cramps | y,y,y,y | dis-symp |
| 狂犬病,呼吸困难 | Rabies, difficulty breathing | m,y,y,y | dis-symp |
| 狂犬病,流涎 | Rabies, salivation | y,y,y,y | dis-symp |
| 狂犬病,RT-PCR | Rabies, RT-PCR | y,y,y,y | dis-test |
| 狂犬病,病毒抗原检测 | Rabies, viral antigen testing | m,m,m,m | dis-test |
| 狂犬病,核酸阳性 | Rabies, nucleic acid positive | y,y,y,y | dis-result |
| 狂犬病,抗原阳性 | Rabies, antigen positive | y,y,y,y | dis-result |
| 病毒抗原检测,阳性 | Virus antigen test, positive | n,n,n,n | test-result |
| 狂犬病,预防 | Rabies, prevention | y,y,y,y | dis-tre |
| 流感,高热 | Flu, high fever | y,y,y,y | dis-symp |
| 流感,乏力 | Flu, fatigue | y,y,y,y | dis-symp |
| 流感,头痛 | Flu, headache | y,y,y,n | dis-symp |
| 流感,咳嗽 | Flu, cough | y,y,y,y | dis-symp |
| 流感,肌肉酸痛 | Flu, muscle soreness | y,y,y,y | dis-symp |
| 流感,白细胞总数 | Influenza, total white blood cells | y,y,y,y | dis-test |
| 流感,淋巴细胞计数 | Influenza, lymphocyte count | y,y,y,y | dis-test |
| 流感,中性粒细胞 | Influenza, neutrophils | y,y,y,y | dis-test |
| 流感,肌酸激酶 | Influenza, creatine kinase | y,y,y,y | dis-test |
| 流感,AST | Flu, AST | m,m,m,m | dis-test |
| 流感,ALT | Flu, ALT | y,y,y,y | dis-test |
| 流感,肌酸激酶升高 | Influenza, elevated creatine kinase | n,y,n,n | dis-result |
| 流感,白细胞总数减少 | Flu, reduced white blood cells | y,y,y,y | dis-result |
| 流感,淋巴细胞计数减少 | Influenza, reduced lymphocyte count | y,y,y,y | dis-result |
| 流感,核酸检测 | Influenza, nucleic acid testing | y,y,y,y | dis-test |
| 流感,核酸检测阳性 | Flu, nucleic acid test positive | n,n,y,y | dis-result |
| 核酸检测,阳性 | Nucleic acid test, positive | y,y,y,y | test-result |
| 肌酸激酶,升高 | Creatine kinase, elevated | y,y,y,y | test-result |
| 白细胞总数,减少 | Total white blood cells, reduced | y,y,y,y | test-result |
| 淋巴细胞计数,减少 | Lymphocyte count, reduced | y,y,y,y | test-result |
| 流感,病毒分离培养 | Influenza, virus isolation and culture | y,y,y,y | dis-test |
| 病毒分离培养,阳性 | Virus isolation and culture, positive | y,y,y,y | test-result |
| 流感,物理降温 | Flu, physical cooling | y,y,y,y | dis-tre |
| 流感,奥司他韦 | Flu, oseltamivir | y,y,y,y | dis-tre |
| 流感,帕拉米韦 | Flu, paramivir | y,y,y,y | dis-tre |
| 流感,莲花清瘟胶囊 | Flu, Lotus Qingwen Capsule | y,y,y,y | dis-tre |
| 胃溃疡,胃痛 | Gastric ulcer | y,y,y,y | dis-symp |
| 胃溃疡,食欲不振 | Gastric ulcer, loss of appetite | y,y,y,y | dis-symp |
| 胃溃疡,腹胀 | Gastric ulcer, bloating | y,y,y,y | dis-symp |
| 胃溃疡,体重减轻 | Gastric ulcer, weight loss | y,y,y,y | dis-symp |
| 胃溃疡,胃出血 | Gastric ulcer, stomach bleeding | y,y,y,y | dis-symp |
| 胃溃疡,黑便 | Gastric ulcer, melena | y,y,y,y | dis-symp |
| 胃溃疡,胃镜检查 | Gastric ulcer, gastroscopy | y,y,y,y | dis-test |
| 胃溃疡,X光检查 | Gastric ulcer, x-ray | y,y,y,y | dis-test |
| 胃溃疡,Hp感染 | Gastric ulcer, Hp infection | y,y,y,y | dis-test |
| 胃溃疡,全血细胞计数 | Gastric ulcer, whole blood count | y,y,y,y | dis-test |
| 胃溃疡,抑酸 | Gastric ulcer, acid suppression | y,y,y,y | dis-tre |
| 全血细胞计数,贫血 | Whole blood cell count, anemia | y,y,y,y | test-result |
| 胃溃疡,Hp阳性 | Gastric ulcer, Hp positive | m,m,m,m | dis-result |
| Hp感染测试,阳性 | Hp infection test, positive | y,y,y,y | test-result |
| 青光眼,眼胀 | Glaucoma | y,y,y,y | dis-symp |
| 青光眼,头痛 | Glaucoma, headache | y,y,y,y | dis-symp |
| 青光眼,恶心 | Glaucoma, nausea | y,y,y,y | dis-symp |
| 青光眼,雾视 | Glaucoma | y,y,y,y | dis-symp |
| 青光眼,虹视 | Glaucoma | y,y,y,y | dis-symp |
| 青光眼,眼压 | Glaucoma, intraocular pressure | y,y,y,y | dis-test |
| 青光眼,视野测试 | Glaucoma, visual field test | y,y,y,y | dis-test |
| 青光眼,眼压高 | Glaucoma, high intraocular pressure | y,y,y,y | dis-result |
| 青光眼,视野缩小 | Glaucoma with reduced field of view | y,y,y,y | dis-result |
| 眼压,高 | Intraocular pressure, high | y,y,y,y | test-result |
| 青光眼,滴眼液 | Glaucoma, eye drops | y,y,y,y | dis-tre |
| 青光眼,降眼压 | Glaucoma, lowering intraocular pressure | y,y,y,y | dis-tre |
| 青光眼,激光治疗 | Glaucoma, laser treatment | y,y,y,y | dis-tre |
| 烧伤,红斑 | Burns, erythema | y,y,y,y | dis-symp |
| 烧伤,红肿 | Burns, redness | y,y,y,y | dis-symp |
| 烧伤,水疱 | Burns, blisters | y,y,y,y | dis-symp |
| 烧伤,皮肤破损 | Burns, broken skin | y,y,y,y | dis-symp |
| 烧伤,疼痛 | Burns, pain | y,y,y,y | dis-symp |
| 烧伤,抗感染 | Burns, anti-infective | n,n,n,n | dis-tre |
| 烧伤,破伤风霉素 | Burns, tetanus | y,y,y,y | dis-tre |
| 烧伤,补液 | Burns | y,y,y,y | dis-tre |
| 哮喘,喘息 | Asthma, wheezing | y,y,y,y | dis-symp |
| 哮喘,气急 | Asthma, shortness of breath | y,y,y,y | dis-symp |
| 哮喘,胸闷 | Asthma, chest tightness | y,y,y,y | dis-symp |
| 哮喘,咳嗽 | Asthma, cough | y,y,y,y | dis-symp |
| 哮喘,胸部X光 | Asthma, chest x-ray | y,y,y,y | dis-test |
| 哮喘,肺功能 | Asthma, lung function | y,y,y,y | dis-test |
| 哮喘,氧饱和度 | Asthma, oxygen saturation | y,y,y,y | dis-test |
| 哮喘,支气管激发 | Asthma, bronchial challenge | n,y,y,y | dis-test |
| 哮喘,过敏原 | Asthma, allergens | y,y,y,y | dis-test |
| 哮喘,激素治疗 | Asthma, hormone therapy | y,y,y,y | dis-tre |
| 哮喘,茶碱 | Asthma, theophylline | y,y,y,y | dis-tre |
| 哮喘,支气管激发阳性 | Asthma, bronchial challenge positive | m,m,y,y | dis-result |
| 支气管激发,阳性 | Bronchial challenge, positive | m,m,m,m | test-result |
| 恶性淋巴瘤,淋巴结肿大 | Malignant lymphoma, enlarged lymph nodes | y,y,y,y | dis-symp |
| 恶性淋巴瘤,发热盗汗 | Malignant lymphoma, fever and night sweats | y,y,y,y | dis-symp |
| 恶性淋巴瘤,体重下降 | Malignant lymphoma, weight loss | y,y,y,y | dis-symp |
| 恶性淋巴瘤,血常规 | Malignant lymphoma, blood routine | y,y,y,y | dis-symp |
| 恶性淋巴瘤,CT | Malignant lymphoma, CT | y,y,y,y | dis-test |
| 恶性淋巴瘤,MRI | Malignant lymphoma, MRI | y,y,y,y | dis-test |
| 恶性淋巴瘤,组织活检 | Malignant lymphoma, tissue biopsy | y,y,y,y | dis-test |
| 血常规,血红蛋白下降 | Blood routine, decreased hemoglobin | y,y,y,y | test-result |
| 血常规,血小板下降 | Blood routine, thrombocytopenia | y,y,y,y | test-result |
| 血常规,白细胞下降 | Blood routine, white blood cells drop | y,y,y,y | test-result |
| 恶性淋巴瘤,血红蛋白下降 | Malignant lymphoma with decreased hemoglobin | y,y,y,y | dis-result |
| 恶性淋巴瘤,白细胞下降 | Malignant lymphoma with decreased white blood cells | y,y,y,y | dis-result |
| 恶性淋巴瘤,放疗 | Malignant lymphoma, radiotherapy | y,y,y,y | dis-tre |
| 恶性淋巴瘤,MOPP方案 | Malignant lymphoma, MOPP protocol | y,y,y,y | dis-tre |
| 恶性淋巴瘤,伊布替尼 | Malignant lymphoma, Ibutinib | m,m,m,m | dis-tre |
| 恶性淋巴瘤,EPOCH方案 | Malignant lymphoma, EPOCH protocol | y,y,y,y | dis-tre |
| 恶性淋巴瘤,西达本胺 | Malignant lymphoma, sitabine | y,y,y,y | dis-tre |
| 白血病,贫血 | Leukemia, anemia | y,y,y,y | dis-symp |
| 白血病,出血 | Leukemia, bleeding | y,y,y,y | dis-symp |
| 白血病,发热 | Leukemia, fever | y,y,y,y | dis-symp |
| 白血病,感染 | Leukemia, infection | y,y,y,y | dis-symp |
| 白血病,淋巴结肿大 | Leukemia, enlarged lymph nodes | y,y,y,y | dis-symp |
| 白血病,血常规 | Leukemia, blood routine | y,y,y,y | dis-test |
| 白血病,血液生化 | Leukemia, blood biochemistry | y,y,y,y | dis-test |
| 白血病,组织活检 | Leukemia, tissue biopsy | y,y,y,y | dis-test |
| 白血病,输血治疗 | Leukemia, blood transfusion treatment | y,y,y,y | dis-tre |
| 白血病,化疗 | Leukemia, chemotherapy | y,y,y,y | dis-tre |
| 白血病,白细胞升高 | Leukemia, elevated white blood cells | y,y,y,y | dis-result |
| 白血病,血细胞分离 | Leukemia, blood cell separator | m,m,m,m | dis-tre |
| 白血病,免疫治疗 | Leukemia, immunotherapy | y,y,y,y | dis-tre |
| 白血病,造血干细胞移植 | Leukemia, hematopoietic stem cell transplant | y,y,y,y | dis-tre |
| 中耳炎,耳痛 | Otitis media, earache | y,y,y,y | dis-symp |
| 中耳炎,流脓 | Otitis media, pus | y,y,y,y | dis-symp |
| 中耳炎,乏力 | Otitis media, fatigue | y,y,y,y | dis-symp |
| 中耳炎,发热 | Otitis media, fever | y,y,y,y | dis-symp |
| 中耳炎,耳镜检查 | Otitis media, otoscopy | y,y,y,y | dis-test |
| 中耳炎,鼓膜内陷 | Otitis media, tympanic invagination | n,n,n,n | dis-result |
| 中耳炎,鼓室积液 | Otitis media, tympanic fluid | y,y,y,y | dis-result |
| 中耳炎,听力下降 | Otitis media, hearing loss | y,y,y,y | dis-symp |
| 耳镜检查,鼓膜内陷 | Otoscopy, tympanic invagination | n,n,n,n | test-result |
| 耳镜检查,鼓室积液 | Otoscopy, tympanic fluid | y,y,y,y | test-result |
| 中耳炎,抗生素治疗 | Otitis media, antibiotic treatment | y,y,y,y | dis-tre |
| 中耳炎,氯霉素 | Otitis media, chloramphenicol | y,y,y,y | dis-tre |
| 中耳炎,氧氟沙星 | Otitis media, ofloxacin | y,y,y,y | dis-tre |
| 中耳炎,清洗 | Otitis media, cleaning | y,y,y,y | dis-tre |
| 中耳炎,鼓膜修补术 | Otitis media, tympanic membrane repair | n,n,y,y | dis-tre |
| 先心病,心悸 | Congenital heart palpitations | y,y,y,y | dis-symp |
| 先心病,胸闷 | Congenital heart disease, chest tightness | y,y,y,y | dis-symp |
| 先心病,乏力 | Congenital heart disease, weakness | y,y,y,y | dis-symp |
| 先心病,气短 | Congenital heart disease, shortness of breath | y,y,y,y | dis-symp |
| 先心病,心绞痛 | Congenital heart disease, angina pectoris | y,y,y,y | dis-symp |
| 先心病,血饱和度 | Congenital heart disease, blood saturation | y,y,y,y | dis-test |
| 先心病,血饱和度降低 | Congenital heart disease, reduced blood saturation | n,n,n,n | dis-result |
| 血饱和度,降低 | Blood saturation, decreased | y,y,y,y | test-result |
| 先心病,听诊 | Congenital heart disease, auscultation | y,y,y,y | dis-test |
| 先心病,心脏杂音 | Congenital heart disease | y,y,y,y | dis-result |
| 听诊,心脏杂音 | Auscultation, cardiac murmur | y,y,y,y | test-result |
| 先心病,手术治疗 | Congenital heart disease, surgery | y,y,y,y | dis-tre |
| 先心病,介入治疗 | Congenital heart disease, interventional treatment | y,y,y,y | dis-tre |
| 先心病,全血细胞学计数 | Congenital heart disease, whole blood cytology count | n,n,n,n | dis-test |
| 先心病,CT | Congenital heart disease, CT | y,y,y,y | dis-test |
| 先心病,MRI | Congenital heart disease, MRI | y,y,y,y | dis-test |
| 先心病,心电图 | Congenital heart disease | y,y,y,y | dis-test |
| 先心病,房间隔缺损 | Congenital heart disease, atrial septal defect | y,y,y,y | dis-result |
| 心电图,房间隔缺损 | ECG, atrial septal defect | y,y,y,y | test-result |
| 先心病,室间隔缺损 | Congenital heart disease | y,y,y,y | dis-result |
| 脱发,激素水平 | Hair loss, hormone levels | y,y,y,y | dis-test |
| 脱发,皮肤镜 | Hair loss, dermatoscope | y,y,y,y | dis-test |
| 脱发,米诺地尔 | Hair loss, Minoxidil | y,y,y,y | dis-tre |
| 腰椎间盘突出症,腰痛 | Lumbar disc herniation, low back pain | y,y,y,y | dis-symp |
| 腰椎间盘突出症,坐骨神经痛 | Lumbar disc herniation, sciatica | y,y,y,y | dis-symp |
| 腰椎间盘突出症,下肢麻木 | Lumbar disc herniation, numbness of lower limbs | y,y,y,y | dis-symp |
| 腰椎间盘突出症,影像学分析 | Lumbar disc herniation, imaging analysis | y,y,y,y | dis-symp |
| 腰椎间盘突出症,大小便异常 | Lumbar disc herniation, abnormal bowel movements | y,y,y,y | dis-symp |
| 腰椎间盘突出症,体格检查 | Lumbar disc herniation, physical examination | y,y,y,y | dis-test |
| 体格检查,腰椎姿势异常 | Physical examination, abnormal lumbar posture | m,m,y,y | test-result |
| 体格检查,腰部压痛 | Physical Examination, Waist Tenderness | y,y,y,y | test-result |
| 体格检查,腰椎活动受限 | Physical examination, restricted lumbar movement | m,m,m,m | test-result |
| 腰椎间盘突出症,腰椎姿势异常 | Lumbar disc herniation, abnormal posture of the lumbar spine | y,y,y,y | dis-result |
| 腰椎间盘突出症,腰部压痛 | Lumbar disc herniation, lumbar tenderness | y,y,y,y | dis-result |
| 腰椎间盘突出症,腰椎活动受限 | Lumbar disc herniation, restricted lumbar spine motion | y,y,y,y | dis-result |
| 腰椎间盘突出症,神经系统检查 | Lumbar disc herniation, neurological examination | y,y,y,y | dis-test |
| 神经系统检查,皮肤感觉过敏 | Neurological examination, skin irritation | y,y,y,y | test-result |
| 神经系统检查,麻木 | Neurological examination, numbness | y,y,y,y | test-result |
| 神经系统检查,感觉减退 | Nervous system examination, hypothyroidism | y,y,y,y | test-result |
| 腰椎间盘突出症,皮肤感觉过敏 | Lumbar disc herniation, skin hypersensitivity | n,n,n,n | dis-result |
| 腰椎间盘突出症,麻木 | Lumbar disc herniation, numbness | y,y,y,y | dis-result |
| 腰椎间盘突出症,感觉减退 | Lumbar disc herniation, hypoesthesia | n,n,n,n | dis-result |
| 腰椎间盘突出症,腰椎X线 | Lumbar disc herniation | y,y,y,y | dis-test |
| 腰椎间盘突出症,造影检查 | Lumbar disc herniation, angiography | y,y,y,y | dis-test |
| 腰椎间盘突出症,CT | Lumbar disc herniation, CT | y,y,y,y | dis-test |
| 腰椎间盘突出症,MRI | Lumbar disc herniation, MRI | y,y,y,y | dis-test |
| X光,结核 | X-ray, tuberculosis | y,y,y,y | test-result |
| X光,骨病 | X-ray, osteopathy | y,y,y,y | test-result |
| 造影检查,腰椎间盘突出 | Angiography, lumbar disc herniation | y,y,y,y | test-result |
| CT,腰椎间盘突出 | CT, lumbar disc herniation | y,y,y,y | test-result |
| MRI,脊髓神经病损 | MRI, spinal neuropathy | y,y,y,y | test-result |
| MRI,腰椎间盘病变 | MRI, lumbar disc disease | y,y,y,y | test-result |
| 腰椎间盘突出症,脊髓神经病损 | Lumbar disc herniation, spinal neuropathy | m,m,m,m | dis-result |
| 腰椎间盘突出症,卧床休息 | Lumbar disc herniation, bed rest | y,y,y,y | dis-tre |
| 腰椎间盘突出症,腰围保护 | Lumbar disc herniation, waist protection | n,m,y,y | dis-tre |
| 腰椎间盘突出症,牵引治疗 | Lumbar disc herniation, traction treatment | y,y,y,y | dis-tre |
| 腰椎间盘突出症,镇痛药物 | Lumbar disc herniation, analgesics | y,y,y,y | dis-tre |
| 腰椎间盘突出症,手术治疗 | Lumbar disc herniation, surgical treatment | y,y,y,y | dis-tre |
| 牙龈炎,牙龈出血 | Gingivitis, bleeding gums | y,y,y,y | dis-symp |
| 牙龈炎,探诊后出血 | Gingivitis, bleeding after visit | y,y,y,y | dis-test |
| 探诊后出血,阳性 | Bleeding after visit, positive | y,y,y,y | test-result |
| 牙龈炎,洁治术 | Gingivitis, cleansing | y,y,y,y | dis-tre |
| 牙龈炎,过氧化氢 | Gingivitis, hydrogen peroxide | y,y,y,y | dis-tre |
| 慢性鼻炎,鼻塞 | Chronic rhinitis, stuffy nose | y,y,y,y | dis-symp |
| 慢性鼻炎,流涕 | Chronic rhinitis, runny nose | y,y,y,y | dis-symp |
| 慢性鼻炎,头痛 | Chronic rhinitis, headache | y,y,y,y | dis-symp |
| 慢性鼻炎,鼻甲肥大 | Chronic rhinitis, turbinate hypertrophy | y,y,y,y | dis-symp |
| 慢性鼻炎,睡眠障碍 | Chronic rhinitis, sleep disorders | y,y,y,y | dis-symp |
| 慢性鼻炎,鼻内镜检查 | Chronic rhinitis, nasal endoscopy | y,y,y,y | dis-test |
| 鼻内镜检查,鼻黏膜充血 | Nasal endoscopy, nasal congestion | y,y,y,y | test-result |
| 鼻内镜检查,鼻黏膜水肿 | Nasal endoscopy, nasal edema | y,y,y,y | test-result |
| 慢性鼻炎,CT | Chronic rhinitis, CT | y,y,y,y | dis-test |
| CT,鼻黏膜炎性病变 | CT, nasal inflammatory lesions | y,y,y,y | test-result |
| 慢性鼻炎,鼻黏膜炎性病变 | Chronic rhinitis, inflammatory inflammation of the nasal mucosa | y,y,y,y | dis-result |
| 慢性鼻炎,鼻冲洗 | Chronic rhinitis, nasal rinse | y,y,y,y | dis-tre |
| 慢性鼻炎,布地奈德 | Chronic rhinitis, budesonide | y,y,y,y | dis-tre |
| 慢性鼻炎,血管收缩 | Chronic rhinitis, vasoconstriction | y,y,y,y | dis-tre |
| 慢性鼻炎,手术治疗 | Chronic rhinitis, surgical treatment | y,y,y,y | dis-tre |
| 骨折,畸形 | Fracture, deformity | y,y,y,y | dis-symp |
| 骨折,异常活动 | Fracture, abnormal activity | y,y,y,y | dis-symp |
| 骨折,发热 | Fracture, fever | y,y,y,y | dis-symp |
| 骨折,骨擦音 | Bone fracture | y,y,y,y | dis-symp |
| 骨折,X光检查 | Fracture, x-ray | y,y,y,y | dis-test |
| 骨折,CT | Fracture, CT | y,y,y,y | dis-test |
| 骨折,MRI | Fracture, MRI | y,y,y,y | dis-test |
| 骨折,局部肿胀 | Fracture, local swelling | n,n,y,y | dis-symp |
| 骨折,复位 | Fracture, reduction | y,y,y,y | dis-tre |
| 骨折,固定 | Fracture, fixation | y,y,y,y | dis-tre |
| 颈椎病,颈部疼痛 | Cervical spondylosis, neck pain | y,y,y,y | dis-symp |
| 颈椎病,颈部僵硬 | Cervical spondylosis, stiff neck | y,y,y,y | dis-symp |
| 颈椎病,四肢麻木 | Cervical spondylosis, limb numbness | y,y,y,y | dis-symp |
| 颈椎病,头晕 | Cervical spondylosis, dizziness | y,y,y,y | dis-symp |
| 颈椎病,恶心 | Cervical spondylosis, nausea | y,y,y,y | dis-symp |
| 颈椎病,视物模糊 | Cervical spondylosis, blurred vision | y,y,y,y | dis-symp |
| 颈椎病,心跳过速 | Cervical spondylosis, tachycardia | y,y,y,y | dis-symp |
| 颈椎病,X光 | Cervical spondylosis, X-ray | y,y,y,y | dis-test |
| 颈椎病,CT | Cervical Spondylosis, CT | y,y,y,y | dis-test |
| 颈椎病,MRI | Cervical Spondylosis, MRI | y,y,y,y | dis-test |
| X光,畸形 | X-ray, deformed | n,n,n,n | test-result |
| X光,骨刺 | X-ray, bone spur | y,y,y,y | test-result |
| X光,椎间隙狭窄 | X-ray, intervertebral space stenosis | y,y,y,y | test-result |
| CT,锥体病变 | CT, pyramidal disease | y,y,y,y | test-result |
| 颈椎病,消炎镇痛 | Cervical spondylosis, anti-inflammatory and analgesic | y,y,y,y | dis-tre |
| 颈椎病,手术治疗 | Cervical spondylosis, surgical treatment | y,y,y,y | dis-tre |

**Appendix 6. Examples of Evaluated Knowledge Fusion Results**

Here we list some examples of knowledge fusion results

A6.1 Entity Alignment Examples

There are two tables, the first is automatically aligned with a score higher than 0.7 and the second is the ones with a score between 0.3 and 0.7 which are checked by human.

- Bolded items are incorrect ones, for which we add explanations.

| **Merged** | **Merged(en)** | **Merged_to** | **Merged_to(en)** | **explanation** |
| --- | --- | --- | --- | --- |
| 腰椎间盘突出 | Lumbar disc herniation | 腰椎间盘突出症 | Lumbar disc herniation |  |
| X光 | X-ray | X线 | X-ray |  |
| 核磁共振 | Nuclear magnetic resonance | MRI | MRI |  |
| 淋巴癌 | Lymphoma | 恶性淋巴瘤 | Malignant lymphoma |  |
| 出虚汗 | Sweating | 盗汗 | Night sweats |  |
| 放疗 | Radiotherapy | 放射治疗 | Radiation Therapy |  |
| **颅内占位性病变** | **Intracranial space occupying lesion** | **脑瘤** | **Brain tumor** | sub-class, but not same |
| 幽门螺杆菌感染 | Helicobacter pylori infection | Hp感染 | Hp infection |  |
| 白细胞数 | White blood cell count | 白细胞计数 | White blood cell count |  |
| 血常规检查 | Blood test | 血常规 | Blood routine |  |
| 食道癌 | Esophageal cancer | 食管癌 | Esophageal cancer |  |
| 期前收缩 | Prephase contraction | 早搏 | Premature beat |  |
| **阿莫西林** | **Amoxicillin** | **青霉素** | **penicillin** | sub-class, but not same |
| **胃穿孔** | **Gastric perforation** | **消化性溃疡** | **Peptic ulcer** | relation, but not same |
| 恶心 | nausea | 反胃 | nausea |  |
| 血透 | Hemodialysis | 透析 | Dialysis |  |
| **鼻甲肥大** | **Turbinate hypertrophy** | **慢性鼻炎** | **Chronic rhinitis** | related disease |
| 抗体阳性 | Antibody positive | 抗体检测阳性 | Positive antibody test |  |
| 上感 | Feel | 上呼吸道感染 | Upper respiratory tract infection |  |
| **考虑食管中段占位** | **Consider mid-esophageal space** | **食管中段占位** | **Occupation of the middle esophagus** | consideration, and sure |
| 胸部CT平扫 | CT scan of the chest | 胸部CT | Chest CT |  |
| **胸部CT增强** | **Chest CT enhancement** | **胸部CT平扫+增强** | **Chest CT scan + enhancement** | inclusion, but not same |
| **肾输尿管膀胱CT平扫** | **CT scan of renal ureter and bladder** | **膀胱CT** | **Bladder CT** | inclusion, but not same |
| **双肾囊肿** | Double kidney cyst | **左肾囊肿** | Left kidney cyst | different location |
| 雾化吸入治疗 | Nebulization inhalation therapy | 雾化治疗 | Nebulization treatment |  |
| 双肺纹理增多 | Increased lung texture | 两肺纹理增多 | Increased texture of both lungs |  |
| **右侧少量胸腔积液** | **A small amount of right pleural effusion** | **左侧胸腔积液** | **Left pleural effusion** | different location |
| **反流性食管炎** | **Reflux esophagitis** | **胃酸过多** | **Hyperacidity** | related disease |
| **高血压肾病** | **Hypertensive nephropathy** | **慢性肾病** | **Chronic kidney disease** | related, but not same |
| **氯化钾** | **Potassium chloride** | **氯化钠** | **Sodium chloride** | related, but not same |

We have also included a list of entity pairs that have a score between 0.3 and 0.7, and are checked and validated by humans. In the table, “R” means rejecting the two entities to be same node, and “A” means accepting.

| **Entity1** | **English** | **Entity2** | **English** | **HumanJudge** |
| --- | --- | --- | --- | --- |
| 眩晕症 | Vertigo | 前庭病变 | Vestibular disease | R |
| 梅尼尔氏症 | Meniere disease | 美尼尔病 | Meniere disease | A |
| 耳鸣 | tinnitus | 脑鸣 | Naoming (TCM term, tinnitus with vertigo) | R |
| 高血压 | hypertension | 脑供血不足 | Brain Ischemia | R |
| 嗜睡 | Drowsiness | 白天多睡意 | Drowsiness | A |
| 牙龈炎 | Gingivitis | 牙周炎 | Periodontitis | R |
| 荧光透视 | Fluoroscopy | 胸透 | Fluoroscopy | A |
| 腹泻 | diarrhea | 拉肚子 | diarrhea | A |
| 干眼症 | xerophthalmia | 角结膜干燥症 | xerophthalmia | A |
| CT | CT | MRI | MRI | R |
| 占位性病变 | Space-occupying lesion | 肿瘤 | Tumor | R |
| 肺气肿 | emphysema | 气胸 | pneumothorax | R |
| 胸闷 | chest distress | 呼吸困难 | Difficulty breathing | R |
| 腰椎间盘突出 | lumbar herniated disc | 腰椎间盘脱出 | lumbar prolapsed disc | R |
| 发热 | fever | 发烧 | fever | A |
| 半身不遂 | hemiplegia | 偏瘫 | hemiplegia | A |
| 青霉素钠注射液 | Benzylpenicillin Sodium for Injection | 青霉素钠 | Benzylpenicillin Sodium (for Injection) | A |
| 甲状腺功能减退 | hypothyroidism | 甲减 | hypothyroidism | A |
| 关节疼痛 | arthralgia | 韧带损伤 | ligamentous injury | R |
| 消化不良 | dyspepsia | 功能性消化不良 | functional dyspepsia | R |
| 焦虑 | anxiety | 抑郁 | depression | R |
| 卡马西平 | carbamazepine | 卡马西平片 | carbamazepine | A |
| 偏头痛 | migraine | 三叉神经痛 | trigeminal neuralgia | R |
| 非典 | SARS | 非典型性肺炎 | SARS | A |
| 肌肉酸痛 | muscle soreness | 肌肉痉挛 | muscle cramps | R |
| 透析 | dialysis | 血液透析 | HD | R |
| 脑出血 | cerebral hemorrhage | 脑溢血 | cerebral hemorrhage | A |
| 胃肠功能紊乱 | gastrointestinal dysfunction | 胃肠神经官能症 | gastrointestinal dysfunction | A |
| 心悸 | palpitation | 窦性心律失常 | sinus arrhythmia | R |
| 尿路感染 | UTI | 膀胱炎 | cystitis | R |

A6.2 Consistency Resolving Examples

There are two types of conflict: entity classification and relation classification. For entity classification, there may be 1) multiple semantic types are tagged to an entity, or 2) incomplete entity string. For relation classification, there are more cases: 1) multiple semantic types are tagged to a relation, 2) low score for results, which has multiple causes like incomplete entities involved in extracted result, or multiple relations are extracted in a long sentence.

The first table is for entity and second is for relation. In the second table, the relationship has several short names: dis-symp (disease and symptom), dis-tre (disease and treatment/drug), dis-test (disease and test), dis-ter (disease and test_result). The score threshold is 0.7. When a classification result has a score higher than 0.7, it is directly accepted; when a classification result score is between 0.3 and 0.7, it is checked by human. In the tables, we list the scores of conflicted results and operation where ‘auto’ means the problem is resolved automatically and ‘man’ means it is done manually.

1. Entity Classification Resolving

| **Conflict** | **Conflict (EN)** | **Confliction Type** | **Scores** | **Operation** |
| --- | --- | --- | --- | --- |
| 胸腺退化不全(TER,DIS) | Thymic insufficiency (TER, DIS) | Entity Classification | 0.82,0.23 | Solve to TER (auto) |
| 复视虹视(TER,SYM) | Double vision iris (TER, SYM) | Entity Classification | 0.58,0.43 | Solve to SYM (man) |
| 右眼白内障超声乳化(TES,TRE) | Phacoemulsification of right eye cataract (TES, TRE) | Entity Classification | 0.11,0.76 | Solve to TRE (auto) |
| 亚莫利(TES,TRE) | Amori (TES, TRE) | Entity Classification | 0.05,0.91 | Solve to TRE (auto) |
| 怕热多汗(TER,SYM) | Afraid of heat and sweat (TER, SYM) | Entity Classification | 0.27,0.72 | Solve to SYM (auto) |
| 右肺下叶肺气囊(DIS,TER) | Right lower lobe lung balloon (DIS, TER) | Entity Classification | 0.66,0.70 | Solve to TER (man) |
| 肝脏多发占位(DIS,TER) | Multiple Liver Occupations (DIS, TER) | Entity Classification | 0.71,0.56 | Solve to TER (man) |
| 大肠多发息肉(DIS,TER) | Large intestine polyp (DIS, TER) | Entity Classification | 0.22,0.82 | Solve to TER (auto) |
| 颈部淋巴结肿大(TER,SYM) | Cervical lymphadenopathy (TER, SYM) | Entity Classification | 0.15,0.93 | Solve to SYM (auto) |
| 头痛头晕(TES,SYM) | Headache dizziness (TES, SYM) | Entity Classification | 0.05,0.90 | Solve to SYM (auto) |
| 甲氨蝶呤(TES,TRE) | Methotrexate (TES, TRE) | Entity Classification | 0.07,0.79 | Solve to TRE (auto) |
| 盐酸(TES,TRE) | Hydrochloric acid (TES, TRE) | Entity Classification | 0.21,0.38 | Delete this entry  (man) |
| 血尿泡沫尿(DIS,SYM) | Hematuria foam urine (DIS, SYM) | Entity Classification | 0.18,0.86 | Solve to SYM (auto) |
| 移动性浊音(TER,TES) | Mobile dullness (TER, TES) | Entity Classification | 0.33,0.83 | Solve to TES (auto) |
| 伸舌偏斜(TER,SYM) | Tongue deviation (TER, SYM) | Entity Classification | 0.19,0.80 | Solve to SYM (auto) |
| 左侧胸腔积液(DIS,TER) | Left pleural effusion (DIS, TER) | Entity Classification | 0.44,0.87 | Solve to TER (auto) |
| FK506血清药物浓度测定(TER,TES) | FK506 serum drug concentration determination (TER, TES) | Entity Classification | 0.67,0.63 | Solve to TES (man) |
| 切除病肝(DIS,TRE) | Removal of diseased liver (DIS, TRE) | Entity Classification | 0.58,0.64 | Solve to TRE (man) |
| 畏寒(TRE,SYM) | Chills (TRE, SYM) | Entity Classification | 0.20,0.96 | Solve to SYM (auto) |
| 肾病综合征(TES,DIS) | Nephrotic Syndrome (TES, DIS) | Entity Classification | 0.17,0.84 | Solve to DIS (auto) |
| 培门冬酰胺酶(TES) 化疗(TRE) | Asparaginase (TES) chemotherapy (TRE) | Entity Classification  1 entity is split as 2 | 0.31,0.46 | Solve to 'SMILE方案' (man) |
| 骨髓穿刺术(TES,TRE) | Bone Marrow Aspiration (TES, TRE) | Entity Classification | 0.76,0.15 | Solve to TES (auto) |
| 子宫前倾(DIS,TER) | Uterine forward tilt (DIS, TER) | Entity Classification | 0.23,0.85 | Solve to TER (auto) |
| 腹股沟疝(TES,DIS) | Inguinal hernia (TES, DIS) | Entity Classification | 0.19,0.76 | Solve to DIS (auto) |
| 代文胶囊(TES,TRE) | Diovan capsule (TES, TRE) | Entity Classification | 0.18,0.80 | Solve to TRE (auto) |

1. Relation Classification Resolving

| **Conflict** | **Conflict (EN)** | **Confliction Type** | **Scores** | **Operation** |
| --- | --- | --- | --- | --- |
| 急性坏死型胰腺炎-盆腔少量积液 （dis-ter,ter-dis） | Acute necrotizing pancreatitis-small amount of pelvic fluid (Dis-ter, ter-dis) | Relation Classification | 0.72,0.15 | dis-ter(auto) |
| 神经系统检查-明显包块 (dis-ter,low score) | Nervous System Examination-Obvious Mass (dis-ter, low score) | Relation Classification | 0.33 | Remove (man) |
| 淋巴结内异型细胞巢浸润- 乳腺癌or腋下淋巴结继发恶性肿瘤 (2 diseases extracted for a test-result, in 1 sentence) | Nestoid cell infiltration in lymph nodes- Breast cancer or axillary lymph node secondary malignancy (2 diseases extracted for a test-result in 1 sentence) | Relation Classification | 0.19,0.82 | pick disease subject 腋下淋巴结继发恶性肿瘤 axillary lymph node secondary malignancy  (auto) |
| 动态心电图-房早 (tes-dis,tes-ter) | Dynamic ECG-Early Morning (tes-dis, tes-ter) | Relation Classification | 0.72,0.34 | tes-dis (auto) |
| 超声胃镜-间质瘤 (incomplete subject,low score) | Ultrasonic gastroscope-stromal tumor (incomplete subject, low score) | Relation Classification | 0.42 | complete subject: 高位胃体间质瘤 High gastric stromal tumor (man) |
| 乙肝大三阳-胆囊腺肌症 (low score) | Hepatitis B Big Three Yang-Gallbladder Adenomyosis (low score) | Relation Classification | 0.35 | Remove (man) |
| 肝硬化(DIS)-脾静脉增粗迂曲(TER) (low score) | Cirrhosis (DIS)-Splenic Vein Thickening Torsion (TER) (low score) | Relation Classification | 0.38 | remove(man) |
| CT-原多发病灶 (tes-ter,low score, incomplete) | CT-primary multiple lesions (tes-ter, low score, entity is incomplete) | Relation Classification | 0.49 | complete entity: 原多发病灶明显缩小或基本消失 The primary multiple lesions were significantly reduced or disappeared  (man) |
| 全腹剧烈疼痛-胃穿孔or套细胞淋巴瘤 (2 extracted diseases for symp) | Severe abdominal pain-gastric perforation or mantle cell lymphoma (2 extracted diseases for symp) | Relation Classification | 0.88,0.34 | pick disease subject 胃穿孔  gastric perforation  (auto) |
| FVC-[NUM]% (tes-ter,low score, incomplete) | FVC- [NUM]% (tes-ter, low score, incomplete) | Relation Classification | 0.30 | complete subject: FEV1/FVC  (man) |
| 四肢浮肿-直肠癌(sym-dis or ter-dis) | Swollen limbs-rectal cancer (sym-dis or ter-dis) | Relation Classification | 0.79,0.15 | sym-dis  (auto) |
| 咽痛-吞咽困难(sym-dis,low score) | Sore throat-dysphagia (sym-dis, low score) | Relation Classification | 0.56 | confirmed it is correct (man) |
| 口角歪斜-脑梗死(sym-dis,low score) | Squash angle-cerebral infarction (sym-dis, low score) | Relation Classification | 0.36 | confirmed it is correct (man) |
| 体检-肝占位(tes-dis,low score) | Physical examination-liver score (tes-dis, low score) | Relation Classification | 0.68 | removed due to causal relation (man) |
| 体检-尿蛋白阳性(tes-ter,low score) | Physical examination-urinary protein positive (tes-ter, low score) | Relation Classification | 0.55 | remove the entity 'body check'(man) |

**Appendix 7. Examples of QA Results**

Here we list 50 random selected QA results from all the set for QA quality evaluation. All questions and answers are picked from test set of our QA corpus (not used for training). We list here as output is the knowledge fetched from the QA system, not the answer. We have:

- Manually judged whether the knowledge is correct by comparing with the knowledge and the answer in corpus.
- Given explanation and analysis for each answer.
- Used some answering rules for answer improvement.

**Note: only translated questions and answers are listed due to the limit of width of tables.**

| **Question(En)** | **Output(hitted knowledge) and factoid answers(EN)** | **Correctness** | **Explanation** |
| --- | --- | --- | --- |
| Ask about the treatment of cervical syndrome? | <Cervical spine syndrome, **dis-tre**, full rest, exercise, massage> | Correct | direct question |
| Heart murmur is loud. What causes it? Will there be any major illness? | <Mitral stenosis and atresia, **dis-sym**, cardiac murmur> <Aortic stenosis and atresia, **dis-sym**, cardiac murmur> | Correct | it correctly gets target of question |
| What is the compound bone peptide drugs used for? | <Rheumatoid arthritis, **dis-tre**, compound bone peptide> | Correct | it matches 'used' to disease treatment |
| What should I do if I have osteoporosis? | <Osteoporosis, **dis-tre**, vitamin D> A: take vitamin D | Correct | it matches 'what ...do' to disease treatment |
| I have been suffering from back pain when lying on my back. I have no symptoms of sleeping on my side. | <Lumbar muscle strain, **dis-sym**, back pain when supine, usually asymptomatic> A: consider lumbar muscle strain | Correct | no obvious question words. so matches the symptoms in question to disease |
| How to recover from muscle strain while playing football? | <Muscle strain, **dis-tre**, bed rest, restricted activity, hot pack> | Correct | it matches 'how' to disease treatment |
| What's going on on both sides of the nose that is always red and itchy? | <Dry seborrhea, **dis-tre**, clean and hygienic, avoid spicy and greasy, take vitamins> A: consider dry seborrhea,and clean... | Correct | it matches 'what' to disease, and fetches treatment for it.(good feature) |
| Liver function test transaminase 102, others are normal, is there any problem? | <Liver damage, **dis-ter**, elevated alanine aminotransferase> <Hepatitis, **dis-ter**, elevated alanine aminotransferase> <Cirrhosis, **dis-ter**, elevated alanine aminotransferase> <Fat liver, **dis-ter**, elevated alanine aminotransferase> ... returned: many possibilities, please do more test | Correct* | * we added a rule to return many possibilities of disease, and give advice |
| Liver function test results total bilirubin 39 is high, and his complexion is yellow. Do I need further tests? | <HBV, **dis-ter**, HBV DNA test> | Correct | it correctly gets target of question |
| Lethargy. But when I sleep, my nose is always blocked, and my throat is always blocked by sputum. Sometimes I have difficulty breathing, and I feel very restless at night. What can I do to change it? | <Turbinate hypertrophy, **dis-sym**, nasal congestion_sputum_sleepiness_dyspnea> <Rhinitis, **dis-sym**, nasal congestion_dyspnea> returned all possibilities, A: please do more test | Correct* | * we added a rule to return many possibilities of disease, and give advice |
| Sudden discomfort, chills all over, how to treat flu fever? | <Flu, **dis-tre**, cooling, rest, antibacterial, antiviral> | Correct | direct question |
| My dull right lower quadrant pain has been for four or five years, getting heavier and heavier for five or six years. The colonoscopy is a benign polyp. The doctor said that the observation is related to the pain? When the pain is severe, there is a water-like sound when you press it. | <Multiple benign polyps, **dis-tre**, surgical resection>  A: consider multiple benign polyps, and maybe surgical resection is needed. | Correct | it matches symptoms to disease, and fetches treatment for it.(good feature) |
| What medicine to take on high pressure? | <Hypertension, **dis-tre**, irbesartan,enapril,fosinopril> | Correct | direct question |
| Large low-density shadows were seen on the right frontal and parietal occipital lobe, with a CT value of about 10 HU, and the boundaries were clearer; small spotted low-density shadows were seen on the bilateral basal ganglia, with clear boundaries; bilateral dilatation of the ventricles, sulci, and brain The crack was widened and deepened, and the centerline structure was centered. Examination and diagnosis of plain CT scan + three-dimensional reconstruction of the skull: 1. right frontal and parietal occipital lobe cerebral infarction and softening focus formation; 2. bilateral lacunar cerebral infarction. Is this serious? | <Cerebral infarction, **dis-tre**, thrombolytic therapy, medication>  A: consider cerebral infarction, and treatment includes thrombolytic therapy and medication, etc. | Incorrect | The purpose is not to ask treatment, but to ask whether it is serious. The knowledge base and MemNN are hard to get correct purpose and answer this kind of questions. But even for a human doctor, it is also hard. |
| How to lower blood lipids? | <High blood lipids, **dis-tre**, oral statins> | Correct | it matches 'how' to disease treatment |
| Drugs that regulate autonomic nerve function | <Vegetative neurological disorder, **dis-tre**, oryzanol, vitamins> | Correct | direct question |
| Sometimes I wake up in the morning with a lot of headaches, and sometimes I'm so upset at night. | too many memory with low score (no hit) return: please go to hospital to ask doctors | Correct* | various tests are needed |
| My head hurts a bit these days. For five days, my feet are a little weak. | <Vascular neuropathic headache, **dis-sym**, headache, deficiency> <Cerebrovascular spasm, **dis-sym**, headache, weakness> <Excessive fatigue, **dis-sym**, headache, fatigue> return: too many, please have more test | Correct* | * we added a rule to return many possibilities of disease, and give advice |
| There is a little blood in the stool these days, there will be a little pain | <Hemorrhoids, **dis-sym**, Blood in stool,Stool pain> <Anorectal disease, **dis-sym**, blood in stool, stool pain> list all possibilities,A: please do more test | Correct* | * we added a rule to return many possibilities of disease, and give advice |
| My ALT is 54, so the hospital does not allow health certificate, how can we reduce it a little | <Hepatitis, **dis-tre**, liver protection>  A: consider hepatitis, treatment is liver protection | Incorrect  (maybe) | Too abstract judge, useless answer |
| Seek medical attention due to foreign body sensation in the throat. The doctor said that I had chronic pharyngitis and chronic gastritis with erosion! (Has done gastroscopy and laryngoscope). Recently, I suddenly noticed that the right side of my neck is higher than the left! And it hurts to touch! On January 23, 2020, a thyroid b-ultrasound was performed. The doctor bought what was said. Let's say that after the Chinese New Year, let me check the thyroid blood chart! I haven't checked yet! Current symptoms: a foreign body sensation in the throat and a cooing stomach! The neck is still higher on the right than on the left. I don't know what disease I am! | <Chronic pharyngitis, **dis-tes**, laryngoscope> <Erosive gastritis, **dis-tes**, gastroscopy>  A: please do more test like laryngoscope and gastroscopy | Incorrect | The answer has no value much information is contained in the question, so the extracted memories are just with same meaning with the question. |
| Sometimes I do n’t eat for a day, sometimes vomiting, sometimes my body rejects vomiting, my sleep is very bad, my social fear is irritable, my eyes are dark, my head is cranky, and I have had chronic gastritis | <Chronic gastritis, **dis-sym**, vomiting> | Incorrect | Same as the one above: not a satisfactory answer |
| Hello doctor! After the collision of the right forehead! It ’s been three months in the whole forehead and it ’s uncomfortable all day long! How do I treat it? | no matched | Incorrect | 脑震荡, but the KB didn't contain this entry |
| There are a lot of yellow and sticky booger, dry crusts in the right nostril, and it is painful and dry. It was bloodshot with a tissue, and it became crusty again. It lasted for half a month. The online check should be dry. Rhinitis, what medicine should I use? | <Rhinitis, **dis-tre**, surgical treatment,oral Hudan pills,nasal drops> | Incorrect | 1. not exact (too abstract) answer on disease, 2. too many treatments, maybe not all suitable for the actual disease for the patient, but cover the answer |
| How is atypical pneumonia treated? | <SARS, **dis-tre**, antiviral treatment> | Correct | direct question |
| When my brother was 35, he got JE and was in a coma for 80 days. Now two years have passed and his life is barely taken care of himself. Now he mainly has no memory and no ability to control himself. I would like to ask what other treatments are available? | <JE, **dis-tre**, fluid replacement,adrenocortical hormone> | Incorrect | actually the disease is Sequelae of Japanese Encephalitis, not JE itself. |
| What to do if urethral stones cause hydronephrosis? | <Urethral stones, **dis-tre**, lithotripsy> | Correct | it matches 'what to do' to disease treatment |
| Q What are the symptoms of dry eye? | <Dry eye disease, **dis-sym**, dry eyes, blurred vision, eye pain, fear of light> | Correct | direct question |
| It has red acne, looks like what they said about syphilis. It grows below the belly and above the penis, and it is a little red. | <Syphilis, **dis-test**, syphilis antibody check>  A: consider syphilis, do syphilis antibody check | Correct | "looks like" often leads to a test |
| When I tied my daughter's hair, I felt a lump in the neck under her ear, which was hard, and I pushed it to move, but the baby didn't feel the pain. I think this is especially similar to cervical lymph node tuberculosis. | <Lymph node tuberculosis, **dis-sym**, swollen lymph nodes, painless, pushable> | Correct | it correctly matches symptoms expressed in fork language from patient |
| Cerebral ischemic focus, white matter is loose, what is a partially empty saddle? | <Ischemic cerebrovascular disease, **dis-ter**, focal cerebral ischemia, Leukeropenia> | Correct | it matches 'what' to disease |
| Hypothyroidism for more than a year, how to cure | <Hypothyroidism, **dis-tre**, pay attention to rest, Jiayoule> | Correct | it correctly matches 'how to cure' to get treatments |
| I am fifty-two years old, and recently I found that I feel a little dizzy from time to time. What's wrong? | return: many hitted, so recommend to have a test <Anemia, **dis-test**, blood test> <hypertension, **dis-test**, blood pressure> | Correct | asking simple symptoms always have this answer |
| What I want to know now is what are the effective ways to treat asthma? | <Asthma, **dis-tre**, theophylline, preventive treatment> | Correct | different treatments for different cases are put together |
| What should I pay attention to in acute nephritis syndrome? Do I need a kidney puncture? | <Nephritis syndrome, **dis-test**, urine test, renal biopsy> A: Follow the doctor's advice | Incorrect | This question is hard to judge even by a human. it depends on many other tests, so the system can't judge. So we should add the logic. |
| Is tricuspid valve insufficiency a cardiomyopathy? | <Coronary heart disease, **dis-ter**, tricuspid insufficiency> <Congenital heart disease, **dis-ter**, tricuspid insufficiency> <Cardiomyopathy, **dis-ter**, tricuspid insufficiency> return all possibility, and ask to do more tests | Correct* | * we added a rule to return many possibilities of disease, and give advice |
| How can Alzheimer's disease be alleviated? | <Alzheimer's disease, **dis-tre**, brain rehabilitation, thinking exercise> | Correct | it matches 'how' to treatment (simple) |
| Little boy is 6 years old, myocardial enzymes are more than 10,000, ECG is okay, no drop after 8 days of infusion | many low scored results matched, no answer | Incorrect | if test result is a number, it is hard, so we need to add handler for numbers |
| Urine routine: protein (Pro) +/- latent, no obvious symptoms | no hit: no answer | Incorrect | no such knowledge |
| Occult blood +, protein + appears routinely, is it serious? | <Kidney damage, **dis-ter**, urinary occult blood positive, urinary protein positive> <Tumor, **dis-ter**, urinary occult blood positive, urinary protein positive> <Kidney stones, **dis-ter**, urinary occult blood positive, urinary protein positive> ... Too many, so 'need more test' | Correct* | still the rule works |
| Pain under my chest and belly, but a little sick yesterday | <gastropathy, **dis-tre**, oral omeprazole, oral morphin>  A: maybe gastropathy, treat with oral omeprazole, oral morphin | Incorrect | It matches a 'gastropathy', but there are more possibilities |
| Brushing my teeth last night and this morning was nausea, vomiting and bleeding, what happened, my throat didn't hurt | <Chronic pharyngitis, **dis-sym**, nausea while brushing teeth, retching> | Correct | it correctly matches symptoms expressed in fork language from patient |
| Lie on your stomach while standing flat, whether your stomach is bulging? | <Ascites, **dis-test**, X-ray,CT,MRI,puncture examination> | Correct | for questions like 'whether not', it matches test |
| Is leukocytosis a sign of leukemia? | TER "leukocytosis" "hit too many diseases, so answer: more tests are needed " | Correct* | Still the rule works |
| Will the lower extremity femoral head necrosis! What better treatment is there? ? | <Femoral head necrosis, **dis-tre**, femoral head replacement surgery, massage, hot pack> | Correct | direct question |
| I fell down and can straighten, but it hurts to bend | no hit | Incorrect | it can't match symptoms expressed in fork language from patient |
| The physical examination of glutenamine is normal, glutamine 84 is there any problem? | <Liver damage, **dis-test**, five items of hepatitis B, CT, liver color ultrasound> A: consider these tests ... | Correct | from some tests to more tests, good feature |
| Babies over one year old started pulling and throwing up. Then I went to the hospital. The fluid did not improve after a few days of losing. The doctor said that his bowel was blocked. | no hit | Incorrect | it can't match symptoms expressed in fork language from patient |
| I would like to go to the toilet continuously for an hour today, and urinate, I suspect I am acute cystitis, what medicine can I buy to solve it? | <Urinary tract infection, **dis-test**, urine routine, color ultrasound>  A: please do more test like ... | Correct | from some symptoms to more tests, good feature |
| I ate a lot of snow cakes a few days ago. Recently, my gums started to swell and pain. I had small meat packets in my mouth and it was difficult to eat. | <Gingivitis, **dis-tre**, hydrogen peroxide rinse,antibacterial mouthwash>  A: maybe disease is gingivitis, consider the treatment ... | Correct | it gets disease from no question words, and fetches treatment for it.(good feature) |
